# Mutual Exclusion Analysis Shows that DUSP9 Negatively Regulates PD‐L1 Expression and Acts as a Target to Enhance Anti‐PD‐1 Efficacy

**DOI:** 10.1002/advs.202514347

**Published:** 2025-12-17

**Authors:** Yuzhe Hu, Ling Tang, Zheng Kuang, Danyi Huang, Ting Li, Gaofei Yin, Yingyu Chen, Wei Guo, Wenling Han, Pingzhang Wang

**Affiliations:** ^1^ Department of Immunology NHC Key Laboratory of Medical Immunology (Peking University) Medicine Innovation Center for Fundamental Research on Major Immunology‐related Diseases School of Basic Medical Sciences Peking University Health Science Center Beijing 100191 China; ^2^ Peking University Center for Human Disease Genomics No. 38 Xueyuan Road Beijing 100191 China; ^3^ Department of Otorhinolaryngology Head and Neck Surgery Beijing Tongren Hospital, Capital Medical University, Key Laboratory of Otolaryngology Head and Neck Surgery (Capital Medical University) Ministry of Education Beijing 100730 China

**Keywords:** combination therapy, immune checkpoint blockade (ICB), mutual exclusion interference (MEi) strategy, PD‐L1, DUSP9, STAT3

## Abstract

The expression level of PD‐L1 is one of the most widely used predictive markers of immune checkpoint blockade (ICB) efficacy in the clinic, suggesting the importance of regulating PD‐L1 expression. However, no published reports have addressed the systematic exploration of the regulation of immune checkpoint molecules from the perspective of mutual exclusion (ME) in gene expression. The ME analysis, based on gene plasticity, provides a novel perspective on the intergenic regulatory paradigm. Here, multiple negative regulators of PD‐L1 expression are identified, and dual‐specificity phosphatase 9 (DUSP9) is selected for intensive study. DUSP9 negatively regulates PD‐L1 expression in multiple tumor cells, and mechanistically, DUSP9 dephosphorylates STAT3 to mediate the inhibitory role. In syngeneic tumor models, the combination of DUSP9 targeting and PD‐1 antibody can enhance therapeutic sensitivity. The clinical data demonstrated that elevated DUSP9 expression is correlated with diminished PD‐1/PD‐L1 antibody response rates. Consequently, DUSP9 emerges as a promising target for enhancing treatment response in combination with PD‐1 antibody, and functions as a potential marker for predicting the efficacy of tumor immunotherapy. This research demonstrates an efficient method for identifying negative regulators of highly plastic genes (HPGs), which can predict immunotherapy responses and identify new targets for combination therapy with ICB.

## Graphical abstract

A mutually exclusive screening system is established to identify negative regulators of highly plastic genes. Dual specificity phosphatase (DUSP9) is a novel negative regulatory molecule of PD‐L1 by dephosphorylating STAT3, and acts as a target molecule in combination with PD‐1 antibody for tumor immunotherapy and a new clinical biomarker for predicting responsiveness to ICB therapy.

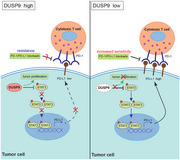

## 1 Introduction

Cancer immunotherapies have made significant advances in the clinical treatment of cancer by enhancing anti‐tumor immune responses. Immune checkpoint blockade (ICB) therapies, particularly antibodies targeting programmed cell death 1 (PD‐1) and PD‐1 ligand 1 (PD‐L1, also known as CD274), have demonstrated durable clinical responses in multiple tumor types.^[^ ^1^, ^2^, ^3^, ^4^ ^]^ However, the average response rates are still low at 20–30%.^[^ ^5^, ^6^, ^7^ ^]^ Therefore, it is crucial to elucidate the mechanisms of resistance to ICB and to develop new strategies to improve the clinical outcome of patients.

The efficacy of tumor immunotherapy is influenced by many factors, including the host, tumor, and tumor microenvironment. PD‐L1 expression is one of the most widely used predictive biomarkers in cancer patients and has been positively correlated with ICB efficacy.^[^ ^8^, ^9^, ^10^, ^11^ ^]^ Patients with high PD‐L1 expression have benefited more from anti‐PD‐1/PD‐L1 therapies. Notably, an increase in PD‐L1 expression during treatment better reflects the efficacy of ICB than pre‐treatment PD‐L1 levels.^[^ ^12^, ^13^, ^14^ ^]^ Therefore, exploring the regulatory mechanism of PD‐L1 expression may help to predict the response to immunotherapy. Although numerous studies have examined the regulation of PD‐L1 expression at different levels,^[^ ^15^ ^]^ research from the perspective of expression exclusivity is lacking. Mutual exclusion (ME) means that two events cannot occur at the same time or simultaneously. Therefore, when PD‐L1 is highly expressed, its ME molecules exhibit extremely low or undetectable expression.^[^ ^16^ ^]^ Conversely, when these ME molecules are highly expressed, PD‐L1 is destined to be expressed at low or nonexistent levels. The current study assumes that high expression of PD‐L1 ME molecules may impact the response to anti‐PD‐1/PD‐L1 immunotherapy. Conversely, disrupting the function of these molecules is expected to increase sensitivity to PD‐1/PD‐L1 antibody therapy.

Traditional Pearson and Spearman correlations are not suitable for analyzing mutually exclusive expressions because the increasing strength of the negative correlation also increases the co‐existence rather than the mutual exclusion.^[^ ^16^, ^17^ ^]^ Therefore, we established the virtual sorting method combined with cosine similarity for ME analysis.^[^ ^16^, ^18^ ^]^ Virtual sorting, also known as in silico sorting, refers to analyzing omic data samples using virtual methods rather than physical objects. Virtual sorting has been used to analyze differentially expressed genes (DEGs) based on changes in gene expression rank.^[^ ^16^, ^18^ ^]^ Cosine similarity is used to measure the angle between two vectors representing the expression of mutually exclusive gene pairs. When the two vectors form a 90‐degree angle, the cosine similarity approaches 0, which indicates mutual exclusivity. All of these analyses fall under gene plasticity analysis in our studies.

The dynamic expression of genes such as PD‐L1 was termed expressional gene plasticity, which refers to the change and variability of gene expression in response to conditions.^[^ ^19^ ^]^ The central dogma describes the flow direction of genetic information transmission, while gene plasticity reflects the magnitude of changes to genetic information during transmission. The entire collection of plasticity constitutes the plasticitome, which includes, but is not limited to, all plastic genes. Gene plasticity can be assessed quantitatively using the gene plasticity score, which reflects changes in the percentile rank of gene expression. Thus, genes can be classified into two main types: high and low plasticity.^[^ ^17^, ^18^, ^19^ ^]^ Biologically, high plasticity indicates that genes are highly expressed to produce a large number of products under some conditions, but show no or extremely low expression under others, and low plasticity indicates that genes generally show broadly high expression or low/no expression under different conditions. Gene plasticity analysis has been used to evaluate marker molecules, discover immune cell subsets and novel phenotypes, stratify cellular phenotypes, and study gene regulation, including co‐phenotypes and mutually exclusive phenotypes.^[^ ^17^, ^18^, ^19^, ^20^, ^21^, ^22^, ^23^, ^24^ ^]^

The highly inducible expression characteristics of PD‐L1 in the tumor microenvironment, such as in response to IFN‐γ stimulation, suggest that PD‐L1 is a highly plastic molecule. This provides an opportunity to identify ME molecules of PD‐L1 in a cell‐type‐specific manner.^[^ ^16^ ^]^ However, due to the low expression level and plasticity of PD‐L1 observed in some tumor cells, ME analysis around the source gene (PD‐L1) is impossible. For example, PD‐L1 exhibits low gene expression and plasticity in tumor cell lines such as HepG2, Huh7, K562, MCF7, and T47D,^[^ ^16^ ^]^ making it unsuitable for ME analysis in these cells. On the other hand, if the target gene exhibits low plasticity in specific cells, it will not be selected as a PD‐L1 ME candidate molecule through ME analysis in those cells. For example, METTL7B (methyltransferase like 7B) is mutually exclusive with PD‐L1 expression, yet it is minimally expressed in H1299, Jurkat, LNCaP, and SKBR3 tumor cells.^[^ ^16^ ^]^ Therefore, METTL7B does not appear in the ME gene list for PD‐L1 in these cells.^[^ ^16^ ^]^ These phenomena are called unilateral exclusion (see below), which means that only one of the source or target genes is highly plastic, while the other is a low‐plasticity molecule with low expression. This complicates the analysis of mutual exclusion.

Therefore, in this study, we pooled 600 RNA sequencing samples from various cell phenotypes to increase the expression gene plasticity of PD‐L1 and overcome the sensitivity limitations of ME analysis caused by unilateral exclusion. Using the method described in this article, we identified a series of PD‐L1 ME candidate genes and validated several of them. We focused on DUSP9 (dual‐specificity phosphatase 9) as an ME molecule with PD‐L1 expression across tumor types. Moreover, DUSP9 was identified as a novel negative regulator of PD‐L1. Mechanistically, DUSP9 dephosphorylates STAT3, thereby inhibiting PD‐L1 expression. Tumors with DUSP9 knockdown were more sensitive to PD‐1 antibody treatment, suggesting that DUSP9 may be a promising target for combination therapy with PD‐1/PD‐L1 antibodies to enhance treatment response. Additionally, high DUSP9 expression is associated with resistance to anti‐PD‐1/PD‐L1 treatment and may serve as a clinical biomarker for non‐response.

Therefore, the current study on the ME analysis of PD‐L1 reveals new opportunities for identifying responsive marker molecules and potential targets for ICB combination immunotherapy. Additionally, the ME analysis in this study provides new insights into the rapid and efficient identification of negative regulation.

## 2 Results

### 2.1 Identification of Mutually Exclusive Genes of PD‐L1 Based on Mixed Cell Phenotypes

**Figure 1:**
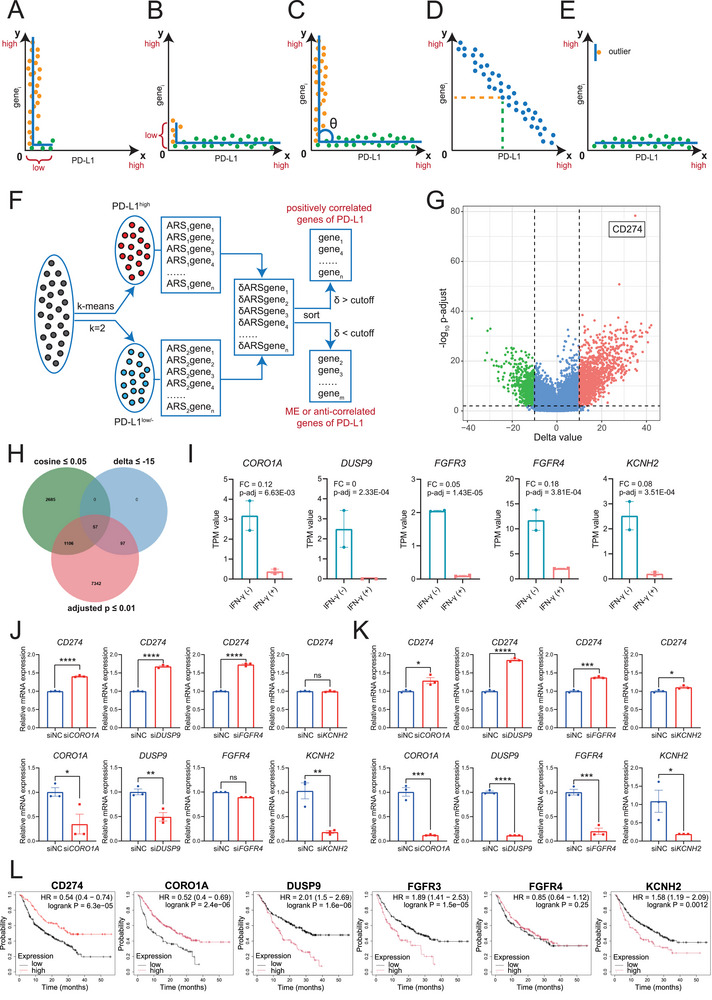
Identification of the mutually exclusive molecules of PD‐L1 expression. A,B) A model showing unilateral exclusion due to the low plasticity of PD‐L1 (A) and any of the other genes (B), such as gene_i_. Each point represents a sample. C) A model of bilateral exclusion is used in this study. D) Linear negative correlation comprises co‐expression with relatively high expression levels in the indicated samples. E) Cosine similarity is sensitive to outlier values. F) The workflow chart shows the PD‐L1 mutually exclusive genes screening by virtual sorting. G) The volcano plot shows the delta value and adjusted *p* value of differential expression based on virtual sorting of PD‐L1^high^ and PD‐L1^low/−^ samples. H) The Venn diagram shows the number of genes that intersect across three screening conditions: cosine ≤ 0.05, delta ≤ ‐15, and adjusted *p* value ≤ 0.01. I) The expression of CORO1A, DUSP9, FGFR3, FGFR4, and KCNH2 was examined using A549 RNA‐Seq data. Data are shown as mean ± SEM; n = 2; adjusted *p* values were calculated by DESeq2. J,K) The expression of PD‐L1 with the indicated gene knockdown was detected by qPCR in A549 (J) and Huh7 (K) cells. Data are shown as mean ± SEM; n = 3; *p* values were determined by Student's t‐test; ns, not significant; ^*^, *p* < 0.05; ^**^, *p* < 0.01; ^***^, *p* < 0.001; ^****,^ *p* < 0.0001. L) Survival analysis of patients who received anti‐PD‐1 therapy, grouped by CD274 and the indicated gene expression, as determined by Kaplan‐Meier Plotter (http://kmplot.com/analysis/).

To avoid omitting information caused by the unilateral exclusion of source and target genes (**Figure** **1**A,B), this study interrogated 12 000 RNA sequencing (RNA‐Seq) samples from the Gene Expression Omnibus (GEO) database. The samples were primarily from primary cells and cell lines. To simplify the calculations, 600 samples (5%) were randomly selected, and their raw data were downloaded for further analysis. Unlike our previous analysis of expressional gene plasticity, which used a single cell type,^[^ ^16^, ^18^ ^]^ these samples come from different cell types and represent a mixed cell phenotype. In the current analysis, the average gene plasticity score for all 19 961 protein‐coding genes is 40, whereas the average score for all single tumor cell types is approximately 25.^[^ ^16^ ^]^ This suggests a significant increase in gene plasticity, indicating plastic or dynamic gene expression across different cell types. PD‐L1's gene plasticity score is 64.88, which is ≈1.62 times higher than the average score of all genes. Therefore, PD‐L1 is a highly plastic gene that can be used for further analyses.

As shown in Figure 1C, the bilateral mutual exclusion (ME) pattern is nonlinear rather than linear. ME describes a scenario where high expression of gene A (e.g., PD‐L1) and high expression of gene B (e.g., gene_i_) rarely or never occur in the same sample (Figure 1C), whereas negative correlation (linear model) can still allow for co‐expression at relatively high expression levels in some samples (Figure 1D). This suggests that linear models are not appropriate for analyzing mutual exclusions, a finding that is supported by our previous studies.^[^ ^16^, ^17^ ^]^ Although cosine similarity can be used for mutual exclusion analysis, it only represents the direction of change in the angle between two vectors (Figure 1C). For example, of the 19961 genes, 3848 have a cosine similarity of less than 0.05 with CD274 (Table S1, Supporting Information). Many noise signals and abnormal values from low‐plasticity genes cause the cosine similarity to approach 0, indicating a 90‐degree angle (Figure 1E). Therefore, cosine similarity does not represent the magnitude of changes in expression levels (i.e., plasticity) between genes. This makes it difficult to identify true ME events.

To address this issue, we introduced a virtual sorting method (Figure 1F) and used the k‐means method (k = 2) to divide the samples into two groups based on PD‐L1 expression: PD‐L1^high^ and PD‐L1^low/−^. We identified differentially expressed genes (DEGs) by analyzing the change in percentile rank (delta value) of all genes in the two groups of samples (see Experimental Section). As shown in Figure 1G, large delta values indicate significant differences in gene expression based on the delta volcano plot, which shows the delta values instead of the fold changes (FC) on the *x*‐axis, unlike the traditional volcano (or called FC volcano) plot. Using strict filtering conditions (delta ≤‐15 and adjusted *p* ≤ 0.01), we screened 154 candidate genes that were mutually exclusive with PD‐L1 expression. These genes were further filtered using a cosine similarity threshold of 0.05, yielding a final set of 57 target genes (Figure 1H; Table S1, Supporting Information). Additionally, virtual sorting analyses revealed that PD‐L1 was a significantly downregulated and differentially expressed gene when these 57 genes were used as the source (Table S1, Supporting Information). Further analyses revealed distinct expression patterns and plasticity of these 57 genes in cancer cell lines (Figure S1, Supporting Information). Additionally, a negative correlation between these genes and PD‐L1 expression was demonstrated using data from the Cancer Cell Line Encyclopedia (CCLE)^[^ ^25^ ^]^ (Figure S2, Supporting Information) and the present dataset (Figure S3, Supporting Information). Thirty‐seven of these genes could not be filtered out based on cell‐type‐specific analysis in our previous study.^[^ ^16^ ^]^ Therefore, increasing gene plasticity by mixing cell phenotypes is an important strategy for mutual exclusion analysis.

### 2.2 Mutual Exclusion is an Efficient and Convenient Method for Identifying Negative Regulators of PD‐L1 Expression

Although ME does not imply a causal relationship, our previous studies have shown that ME indicates negative regulation of gene expression.^[^ ^16^, ^23^, ^24^ ^]^ To further explore the potential regulation of PD‐L1 expression by these genes, we used A549 cells as an initial screening model. A total of 1 × 10⁵ cultured A549 cells were harvested before and after 24 h stimulation with 50 ng mL^−1^ IFN‐γ. Two replicates were performed for each condition, and the cells were used for RNA sequencing. We hypothesized that genes mutually exclusive with PD‐L1 would significantly decrease in response to a dramatic increase in PD‐L1 expression.

As expected, PD‐L1 expression increased significantly after IFN‐γ stimulation, with a fold change of 16.85 (adjusted *p* value = 2.15E‐12). Twenty‐four of the 57 genes were minimally expressed in A549 cells, with TPM values below 0.5 before and after stimulation, and showed no significant differential expression. Of the remaining 33 genes, 26 were down‐regulated after stimulation. However, only five of these changes were statistically significant: FGFR3 (fibroblast growth factor receptor 3), FGFR4, KCNH2 (potassium voltage‐gated channel subfamily H member 2), DUSP9 (dual specificity phosphatase 9), and CORO1A (coronin 1A) (Figure 1I). The other seven genes were upregulated, but only one gene, S100P (S100 calcium binding protein P), had a greater than twofold change and was statistically significant (*p* = 2.57E‐10). The reason for the upregulation of the gene after IFN‐γ stimulation, such as the effect of stimulation time and protein levels, remains to be studied.

Of the five significantly downregulated genes, FGFR3 was identified as a negative regulator of PD‐L1 expression.^[^ ^26^ ^]^ FGFR3 knockdown with siRNA promoted PD‐L1 upregulation in bladder cancer cells.^[^ ^26^ ^]^ Therefore, we only validated the regulatory potential of the remaining four genes on PD‐L1 expression. In independent experiments with A549 and Huh7 cells, we used real‐time quantitative PCR (RT‐qPCR) to detect PD‐L1 expression after knocking down FGFR4, KCNH2, DUSP9, and CORO1A mRNA expression, respectively. As shown in Figure 1J,K, knocking down CORO1A and DUSP9 expression significantly increased IFN‐γ‐induced PD‐L1 expression in both A549 (Figure 1J) and Huh7 (Figure 1K) cells. Although FGFR4 knockdown efficiency was relatively low in A549 cells compared to Huh7 cells, it could still promote IFN‐γ‐induced PD‐L1 expression. Interestingly, knocking down KCNH2 expression significantly increased PD‐L1 expression only in Huh7 cells, not A549 cells (Figure 1J,K). These results suggest that the negative regulatory effect of mutually exclusive genes may depend on the cell type, though more evidence is needed.

Next, we examined the clinical significance of the aforementioned five genes in patients receiving anti‐PD‐1 therapy (Figure 1L). PD‐L1 (CD274) served as a positive control and was significantly correlated with a better clinical prognosis, meaning patients with high CD274 expression had a higher overall survival rate after anti‐PD‐1 therapy. This finding is consistent with previous reports.^[^ ^8^ ^]^ High expression of KCNH2, DUSP9, and FGFR3 corresponded to a poorer prognosis, whereas high CORO1A expression corresponded to a better prognosis (Figure 1L). This may be related to CORO1A's more complex expression profile in the tumor microenvironment, particularly its high expression in immune cells.^[^ ^27^ ^]^ However, no significant prognosis was observed based on FGFR4 expression. DUSP9 had the highest hazard ratio (HR = 2.01) and was the most significant (*p* = 1.6e‐06). Additionally, knocking down DUSP9 expression significantly increased PD‐L1 expression in both A549 and Huh7 cells (Figure 1J,K). Therefore, we focused on DUSP9 in the next study.

### 2.3 DUSP9 Negatively Regulates PD‐L1 Expression in Multiple Tumor Cells

DUSP9 is specifically highly expressed in the placenta, followed by the kidney, with no or low expression in the vast majority of normal tissues (Figure S4A, Supporting Information). According to data from The Cancer Genome Atlas (TCGA),^[^ ^28^ ^]^ DUSP9 is upregulated in most tumor types, including cervical squamous cell carcinoma (CESC), head and neck squamous cell carcinoma (HNSC), liver hepatocellular carcinoma (LIHC), and lung adenocarcinoma (LUAD) (Figure S4B, Supporting Information). CCLE data showed that DUSP9 is highly expressed in liver cancer cell lines, including Huh7 and HepG2 (Figure S4C, Supporting Information). Our cancer cell line datasets,^[^ ^16^ ^]^ each containing a large number of samples under various conditions, further confirmed that DUSP9 is highly expressed in HepG2 and Huh7 hepatocellular carcinoma cells (Figures S1 and S4D, Supporting Information).

**Figure 2:**
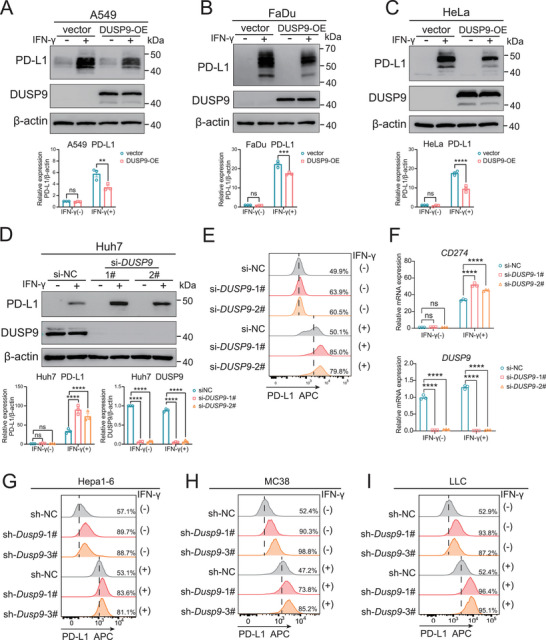
DUSP9 negatively regulates PD‐L1 expression in tumor cells. A–C) The expression of PD‐L1 in DUSP9‐overexpression (DUSP9‐OE) tumor cell lines, including A549 (A), FaDu (B), and HeLa (C), was examined by Western blot. D‐F) The expression of PD‐L1 in DUSP9‐knockdown (DUSP9‐KD) Huh7 cells was detected by Western blot (D), flow cytometry (E), and qPCR (F). G‐I) Flow cytometry results show the expression of PD‐L1 in DUSP9‐KD Hepa1‐6 cells (G), MC38 cells (H), and LLC cells (I). Data are shown as mean ± SEM; n = 3; *p* values were determined by two‐way ANOVA with Sidak's (A–C) or Dunnett's (D, F) post‐hoc test; ns, not significant; ^**^, *p* < 0.01; ^***^, *p* < 0.001; ^****^, *p* < 0.0001.

To investigate the regulatory effects of DUSP9 on PD‐L1 expression, we overexpressed DUSP9 in A549, FaDu, and HeLa cells and treated them with IFN‐γ to induce PD‐L1 expression. As shown in **Figure** **2**A–C, DUSP9 overexpression (OE) attenuated the levels of PD‐L1 upregulation upon IFN‐γ stimulation. Additionally, DUSP9 knockdown (KD) by siRNA in Huh7 cells resulted in increased PD‐L1 expression at the protein and RNA levels in response to IFN‐γ stimulation (Figure 2D). PD‐L1 is the ligand of PD‐1, which mainly locates on cell membranes and plays an immunosuppressive role through the PD‐1/PD‐L1 pathway. Therefore, its membrane surface expression is particularly important. As shown in Figure 2E, flow cytometry confirmed that IFN‐γ‐induced cell surface PD‐L1 expression was also upregulated in DUSP9‐KD Huh7 cells. Furthermore, DUSP9‐KD Huh7 cells also upregulated PD‐L1 at the mRNA level (Figure 2F). These results suggest that DUSP9 negatively regulates PD‐L1 expression in various human tumor cells.

To further investigate the effect of DUSP9 on PD‐L1 expression in mice, we generated lentiviruses containing small hairpin RNAs (shRNAs) that target DUSP9 (sh*Dusp9*) and a negative control (shNC). We then constructed stable cell lines with DUSP9 knockdown. In murine Hepa1‐6 hepatoma cells, all three shRNA sequences efficiently knocked down DUSP9, significantly increasing PD‐L1 expression with or without IFN‐γ treatment (Figure S5A, Supporting Information). Similar results were observed in MC38 colorectal tumor cells (Figure S5B, Supporting Information) and LLC Lewis lung carcinoma cells (Figure S5C, Supporting Information). Flow cytometry confirmed that PD‐L1 expression on the cell surface was increased in DUSP9‐KD Hepa1‐6 (Figure 2G), MC38 (Figure 2H), and LLC (Figure 2I) cells. The mRNA expression of PD‐L1 was also upregulated in DUSP9‐KD Hepa1‐6 (Figure S5D, Supporting Information), MC38 (Figure S5E, Supporting Information), and LLC (Figure S5F, Supporting Information) cells. These results suggest a conservative effect of DUSP9 on PD‐L1 expression.

### 2.4 DUSP9 Reduces PD‐L1 Expression by Catalyzing the Dephosphorylation of STAT3

**Figure 3:**
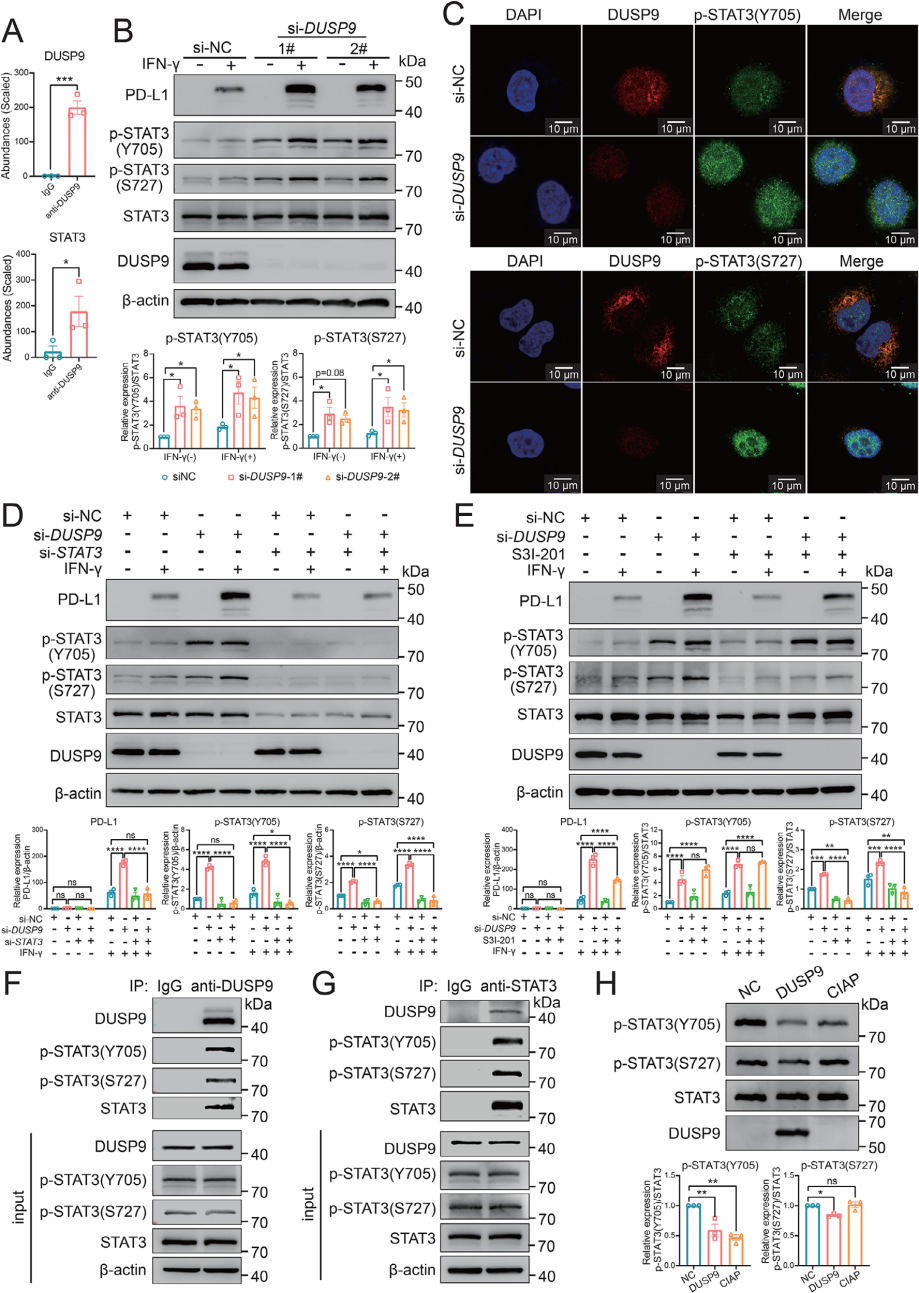
DUSP9 reduces PD‐L1 expression by catalyzing the dephosphorylation of STAT3. A) The abundances of DUSP9 and STAT3 were compared between the IgG and anti‐DUSP9 groups using IP‐MS. B) Western blot results show the expression of PD‐L1 and the phosphorylation of STAT3 in DUSP9‐KD Huh7 cells. C) Immunofluorescence results show the phosphorylation of STAT3 in DUSP9‐KD Huh7 cells. D) The expression of PD‐L1 and the phosphorylation of STAT3 in Huh7 cells after DUSP9 and STAT3 knockdown were examined by Western blot. E) The expression of PD‐L1 and the phosphorylation of STAT3 in DUSP9‐KD Huh7 cells treated with the p‐STAT3‐S727‐specific inhibitor, S3I‐201, were examined by Western blot. F,G) Co‐IP results show the endogenous interaction between DUSP9 and STAT3 when using DUSP9 antibody (F) and STAT3 antibody (G). H) In vitro dephosphorylation assay shows the direct dephosphorylating function of DUSP9 on p‐STAT3 at both Y705 and S727. Positive control: calf intestinal alkaline phosphatase (CIAP). Data are shown as mean ± SEM; n = 3; *p* values were determined by Student's t‐test (A), two‐way ANOVA with Dunnett's post‐hoc test (B), two‐way ANOVA with Sidak's (for PD‐L1) or Tukey's (for p‐STAT3) post‐hoc test (D, E), and one‐way ANOVA with Dunnett's post‐hoc test (H); ns, not significant; ^*^, *p* < 0.05; ^**^, *p* < 0.01; ^***^, *p* < 0.001; ^****^, *p* < 0.0001.

To explore the regulatory mechanism, we performed an immunoprecipitation‐mass spectrometry (IP‐MS) experiment in Huh7 cells to detect DUSP9‐binding proteins. A total of 195 binding proteins were identified (Table S2, Supporting Information). Among them, we noticed that STAT3, a known transcription factor that positively regulates PD‐L1 expression,^[^ ^29^, ^30^ ^]^ showed significantly high abundance in the MS results (**Figure** **3**A). Therefore, we focused on STAT3 for PD‐L1 regulation. As shown in Figure 3B, DUSP9‐KD significantly increased STAT3 phosphorylation at the Y705 and S727 sites regardless of IFN‐γ stimulation. Additionally, the immunofluorescence (IF) staining assay also confirmed that DUSP9‐KD dramatically increased STAT3 phosphorylation at the Y705 and S727 sites, and promoted the entry of phosphorylated STAT3 into the nucleus. The results supported the colocalization of DUSP9 and STAT3 in the cytoplasm (Figure 3C).

To determine whether STAT3 signaling directly mediates the regulatory function of DUSP9 on PD‐L1 expression, we used siRNA to interfere with endogenous STAT3 expression. As STAT3 expression decreased, the PD‐L1 upregulation caused by DUSP9‐KD was almost completely inhibited (Figure 3D). To identify the phosphorylation sites involved in this process, we used the specific STAT3 inhibitor S3I‐201, which selectively inhibits STAT3 phosphorylation at the S727 site.^[^ ^31^ ^]^ The results showed that S3I‐201 partially inhibited the upregulation of PD‐L1 by DUSP9‐KD (Figure 3E). These results confirmed that DUSP9 negatively regulates PD‐L1 by negatively regulating STAT3 phosphorylation at two sites, Y705 and S727.

To determine the endogenous protein‐protein interaction between DUSP9 and phosphorylated STAT3 (p‐STAT3), Co‐immunoprecipitation (Co‐IP) experiments were performed using Huh7 cells. First, DUSP9 was immunoprecipitated using an anti‐DUSP9 antibody. Subsequent Western blot analysis revealed the presence of STAT3 and p‐STAT3 in the immunoprecipitate (Figure 3F). Next, STAT3 was immunoprecipitated using an anti‐STAT3 antibody, which showed that STAT3 could successfully immunoprecipitate DUSP9 (Figure 3G). These consistent Co‐IP results strongly suggest that DUSP9 and STAT3 (or p‐STAT3) interact with each other.

To investigate whether DUSP9 can dephosphorylate STAT3 directly, the recombinant DUSP9 protein was mixed with the HA‐tagged STAT3 protein (HA‐STAT3), which was purified from STAT3‐OE HeLa cells. As shown in Figure 3H, the recombinant DUSP9 protein dephosphorylated both p‐STAT3‐Y705 and p‐STAT3‐S727 directly in vitro, with a particular preference for the Y705 site. These results demonstrate that p‐STAT3 is a novel intracellular substrate of DUSP9 and that dephosphorylated STAT3 mediates decreased PD‐L1 expression in response to IFN‐γ stimulation.

### 2.5 Downregulation of DUSP9 Inhibits Tumor Cell Growth In Vitro and In Vivo

**Figure 4:**
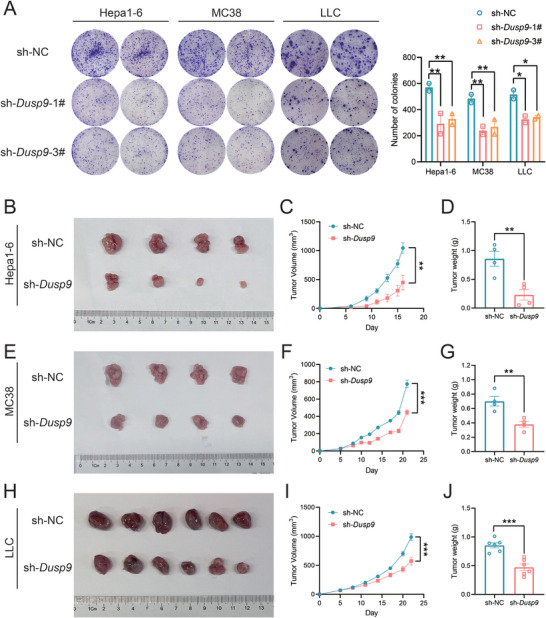
DUSP9 knockdown inhibits tumor growth in vitro and in vivo. A) Colony formation assays were conducted on DUSP9‐KD Hepa1‐6 cells, MC38 cells, and LLC cells. Data are shown as mean ± SEM; n = 2; *p* values were determined by two‐way ANOVA with Dunnett's post‐hoc test; ^*^, *p* < 0.05; ^**^, *p* < 0.01. B–J) An overview of the subcutaneous transplantation tumors of DUSP9‐KD Hepa1‐6 cells (B), MC38 cells (E), and LLC cells (H) in BALB/c nude mice. Tumor growth (C, F, I) and tumor weight (D, G, J) are also shown. Data are shown as mean ± SEM; n = 4 (B–G) or 6 (H–J); *p* values were determined by Student's t‐test; ^**^, *p* < 0.01; ^***^, *p* < 0.001.

Many studies have demonstrated that the upregulation of PD‐L1 enhances the efficacy of ICB therapies.^[^ ^32^, ^33^, ^34^, ^35^ ^]^ The above results showed that human and mouse DUSP9 negatively regulate PD‐L1 expression. Combined with the clinical significance of DUSP9 in anti‐PD‐1 therapy (Figure 1L), it is reasonable to speculate that targeting DUSP9 could improve the response to anti‐PD‐1/PD‐L1 therapy. Since multiple studies have shown that DUSP9 promotes cell proliferation,^[^ ^36^, ^37^, ^38^, ^39^ ^]^ we first examined the impact of targeting DUSP9 on tumor cell growth. Plate colony formation assays of Hepa1‐6, MC38, and LLC cells showed that DUSP9 knockdown inhibited their colony‐forming ability (**Figure** **4**A). For the in vivo experiment, subcutaneous tumor models were established using shNC and sh*Dusp9* tumor cells in BALB/c nude mice. The results showed that DUSP9 knockdown significantly reduced tumor progression in the Hepa1‐6 (Figure 4B–D), MC38 (Figure 4E–G), and LLC (Figure 4H–J) tumor models. These results are consistent with those observed in vitro and suggest that loss of DUSP9 could inhibit tumor growth.

In light of the negative regulation of PD‐L1 expression by DUSP9 in vitro, we examined PD‐L1 cell surface expression by flow cytometry in tumor cells derived from nude mouse tumor tissues (Figure S6A, Supporting Information). PD‐L1 expression was higher in sh*Dusp9* tumor cells than in shNC control tumor cells (Figure S6B, Supporting Information), suggesting that DUSP9 negatively regulates PD‐L1 expression in vivo.

### 2.6 DUSP9 Knockdown Inhibits the Cytotoxic Function of CD8^+^ T Lymphocytes Through PD‐L1

**Figure 5:**
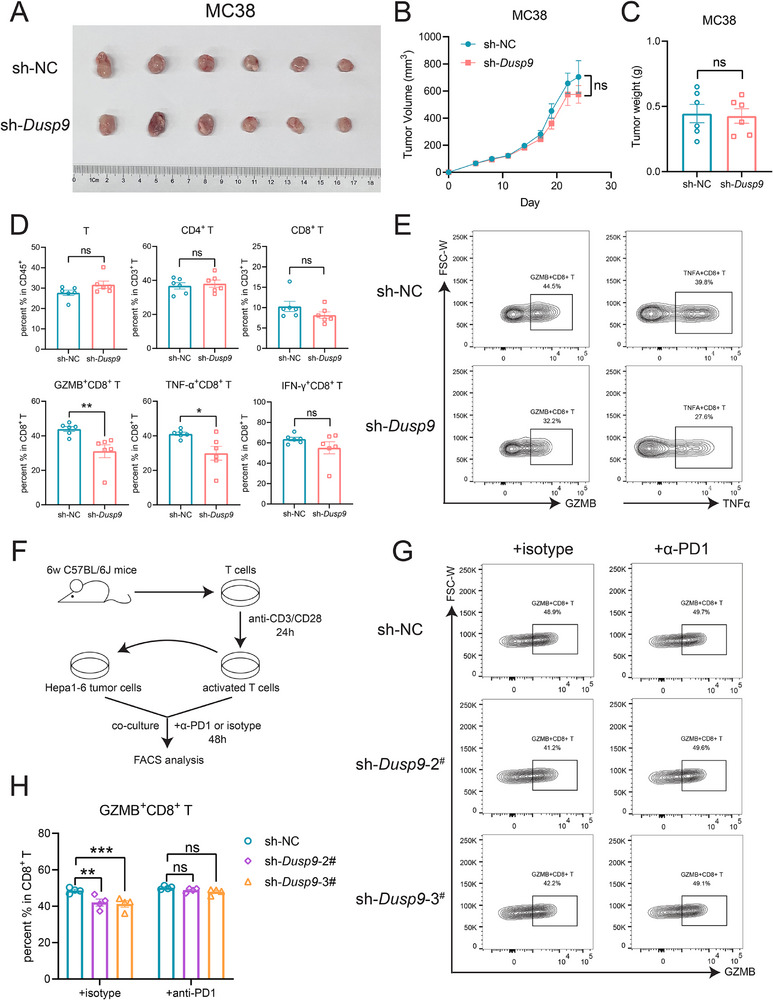
DUSP9 knockdown inhibits cytotoxic CD8^+^ T cells by inducing PD‐L1 expression. A–E) The syngeneic tumor model of DUSP9‐KD MC38 cells in C57BL/6 mice includes the following: (A) tumor overview; (B) tumor growth; (C) tumor weight; (D) statistics on the percentage of tumor‐infiltrating T cell subsets; (E) GZMB^+^CD8^+^ T cell and TNF‐α^+^CD8^+^ T cell subsets detected by flow cytometry. F–H) Co‐culture of primary T cells and DUSP9‐KD Hepa1‐6 cells with anti‐PD‐1 treatment: (F) flowchart; (G) GZMB^+^CD8^+^ T cell subset detected by flow cytometry; (H) statistics on the percentage of the GZMB^+^CD8^+^ T cell subset. Data are shown as mean ± SEM; n = 6 (A–D) or 4 (FH); *p* values were determined by Student's *t*‐test (B–D) or two‐way ANOVA with Dunnett's post‐hoc test (H); ns, not significant; ^*^, *p* < 0.05; ^**^, *p* < 0.01; ^***^, *p* < 0.001.

To investigate the role of DUSP9‐KD in tumor therapy, we inoculated MC38 tumor cells into immunocompetent C57BL/6 mice. Interestingly, contrary to the above findings that DUSP9‐KD attenuated tumor progression and reduced tumor size in immunodeficient mice (Figure 4E–G), we observed no significant difference in tumor growth between mice bearing sh*Dusp9* and shNC tumors (**Figure** **5**A–C). Based on these results, we hypothesized that DUSP9‐KD inhibits T cell function by upregulating PD‐L1, thereby counteracting its inhibitory effect on tumor growth. To test this hypothesis, we detected PD‐L1 cell surface expression in tumor cells and found that PD‐L1 expression was higher in sh*Dusp9* tumor cells than in shNC control tumor cells (Figure S6C, Supporting Information). This finding is consistent with the in vivo results in nude mice. By analyzing immune cell subsets in the tumor microenvironment, we found that the activity of tumor‐infiltrating CD8^+^ T cells, especially GZMB^+^CD8^+^ cytotoxic T cells, was significantly decreased in the sh*Dusp9* group (Figure 5D,E). Similar results were observed in the LLC syngeneic tumor model in immunocompetent C57BL/6 mice (Figure S7A–E, Supporting Information).

To investigate the relationship between PD‐L1 upregulation on tumor cell surfaces and reduced GZMB^+^CD8^+^ cytotoxic T cell activity, we obtained primary T cells from wild‐type C57BL/6 mice. Then, we performed an in vitro co‐culture assay with DUSP9‐KD tumor cells (Figure S7F, Supporting Information). The co‐culture assay revealed that DUSP9 knockdown decreased the percentage of GZMB^+^CD8^+^ cytotoxic T cells (Figure S7G, H, Supporting Information). Next, we used a PD‐1 antibody to block the PD‐1/PD‐L1 axis and used the IgG isotype as a negative control (Figure 5F). The results showed that PD‐1 blockade significantly reversed the reduction in the proportion of cytotoxic T cells compared to the IgG isotype control (Figure 5G,H). These results suggest that the impaired cytotoxic function of CD8^+^ T cells in the DUSP9‐KD tumor group depends on PD‐L1 upregulation on the cell surface.

### 2.7 The Synergistic Effect of DUSP9 Deficiency and PD‐1 Antibody Combination Therapy In Vivo

Based on single‐cell RNA sequencing (scRNA‐Seq) datasets, DUSP9 is predominantly expressed in tumor epithelial cells and is rarely expressed in immune cells and stromal cells, including those in melanoma, hepatocellular carcinoma (HCC), and LUAD (Figure S8, Supporting Information). This specific expression pattern makes DUSP9 a more suitable target for tumor therapy. However, the above results suggest that targeting DUSP9 alone may not suppress tumor growth in immunocompetent mice in vivo. Previous studies have shown, however, that inhibitors targeting negative regulators of PD‐L1 can be used in combination with anti‐PD‐1/PD‐L1 to synergistically inhibit tumor growth.^[^ ^40^, ^41^, ^42^ ^]^ This prompted us to explore combination immunotherapy targeting DUSP9.

**Figure 6:**
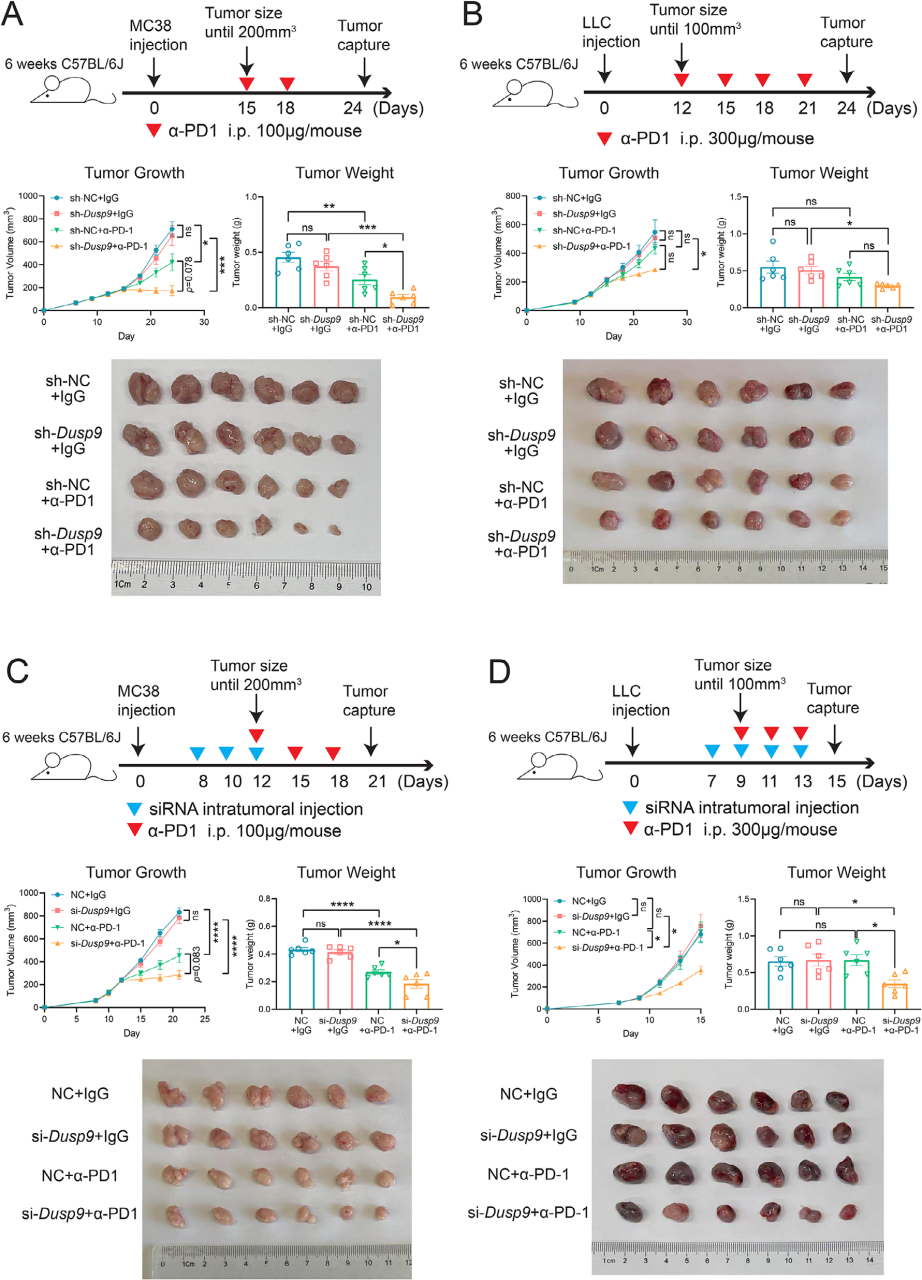
DUSP9 knockdown promotes antitumor efficacy in combination with anti‐PD‐1 therapy. The syngeneic tumor model of C57BL/6 mice was inoculated with various cells and treated as follows: A) DUSP9‐KD MC38 cells and treated with anti‐PD‐1; B) DUSP9‐KD LLC cells and treated with anti‐PD‐1; C) wild‐type MC38 cells and treated with si*Dusp9* and anti‐PD‐1; D) wild‐type LLC cells and treated with si*Dusp9* and anti‐PD‐1. Data are shown as mean ± SEM; n = 6; *p* values were determined by one‐way ANOVA with Sidak's post‐hoc test; ns, not significant; ^*^, *p* < 0.05; ^**^, *p* < 0.01; ^***^, *p* < 0.001; ^****^, *p* < 0.0001.

We inoculated MC38 tumor cells with sh*Dusp9* or shNC into immunocompetent mice. The tumor‐bearing mice were then treated with anti‐PD‐1 or IgG isotype (**Figure** **6**A). We observed no significant difference in tumor volume or weight between the sh*Dusp9* and shNC groups treated with IgG isotype, and anti‐PD‐1 treatment significantly reduced tumor growth compared to IgG isotype (Figure 6A). Importantly, anti‐PD‐1 treatment was more effective in the sh*Dusp9* group than in the shNC group. These results suggest that DUSP9 deficiency combined with PD‐1 blockade exhibits a synergistic effect in vivo (Figure 6A). At the end of treatment, tumor samples were harvested for IF staining. The results showed that anti‐PD‐1 co‐treatment significantly increased tumor‐infiltrating cytotoxic CD8^+^ T cells in vivo (Figure S9A, Supporting Information).

Tumors can be classified into two types according to the infiltration of immune cells in the tumor microenvironment: “hot” tumors, which have abundant immune cell infiltration, and “cold” tumors, which lack immune cell infiltration. “Cold” tumors are less sensitive to immune checkpoint inhibitors.^[^ ^43^ ^]^ To explore whether DUSP9 improves responsiveness to PD‐1 antibody therapy in “cold” tumors, LLC cell lines with stable DUSP9 knockdown were injected into immunocompetent mice and treated with anti‐PD‐1 (Figure 6B). The shNC control group cells were insensitive to PD‐1 antibody treatment, whereas the sh*Dusp9* group cells were responsive (Figure 6B). These results suggest that DUSP9 may serve as a potential therapeutic target in combination with PD‐1 antibody therapy for “cold” tumors. Flow cytometry analysis of infiltrating T cells in tumor tissue revealed that PD‐1 antibody treatment increased GZMB^+^CD8^+^ and IFN‐γ^+^CD8^+^ T cells in the sh*Dusp9* group (Figure S9B, Supporting Information). This demonstrates that LLC tumors with DUSP9 targeting are more sensitive to PD‐1 antibody treatment.

In order to better fit into the clinical applications, we synthesized an siRNA targeting the mouse *Dusp9* gene and introduced it using an in vivo siRNA transfection reagent. Wild‐type MC38 cells were injected subcutaneously and received *Dusp9* siRNA and PD‐1 antibody treatment, respectively (Figure 6C). The results showed that PD‐1 antibody treatment significantly reduced tumor growth, while the group receiving both siRNA and anti‐PD‐1 treatment had an even greater reduction in tumor size and weight (Figure 6C). These results indicate that targeting DUSP9 and PD‐1 antibody blockade have a synergistic effect in vivo. Flow cytometry analysis of infiltrating T cells in tumor tissue revealed that PD‐1 antibody treatment reduced the proportion of CD4^+^ T cells and increased the proportion of CD8^+^ T cells, particularly the cytotoxic GZMB^+^CD8^+^ T cells and IFN‐γ^+^CD8^+^ T cells (Figure S9C, Supporting Information).

To investigate whether siRNA targeting *Dusp9* can enhance responsiveness to PD‐1 antibody therapy in “cold” tumors in mice, we used wild‐type LLC cells to grow subcutaneous tumors and administered *Dusp9* siRNA and PD‐1 antibody treatment (Figure 6D). There was no significant difference in tumor growth between the two groups treated with IgG isotype controls. The control group, which did not receive siRNA treatment, showed no response to PD‐1 antibody therapy. However, the experimental group, which received both *Dusp9*‐targeting siRNA and PD‐1 antibody therapy, experienced reduced tumor growth, size, and weight. This indicates that targeting *Dusp9* can enhance the responsiveness of “cold” tumors to PD‐1 antibody blockade (Figure 6D). Flow cytometry analysis of infiltrating T cells in tumor tissues revealed that the combination of *Dusp9* siRNA and PD‐1 antibody treatment significantly increased the proportion of T cell infiltration, particularly of GZMB^+^CD8^+^ and IFN‐γ^+^CD8^+^ cytotoxic T cells, in the tumor microenvironment (Figure S9D, Supporting Information). These results confirm the therapeutic potential of targeting DUSP9 in combination with PD‐1 antibody therapy.

### 2.8 DUSP9 is a Potential Clinical Biomarker for Predicting Immunotherapy Response

**Figure 7:**
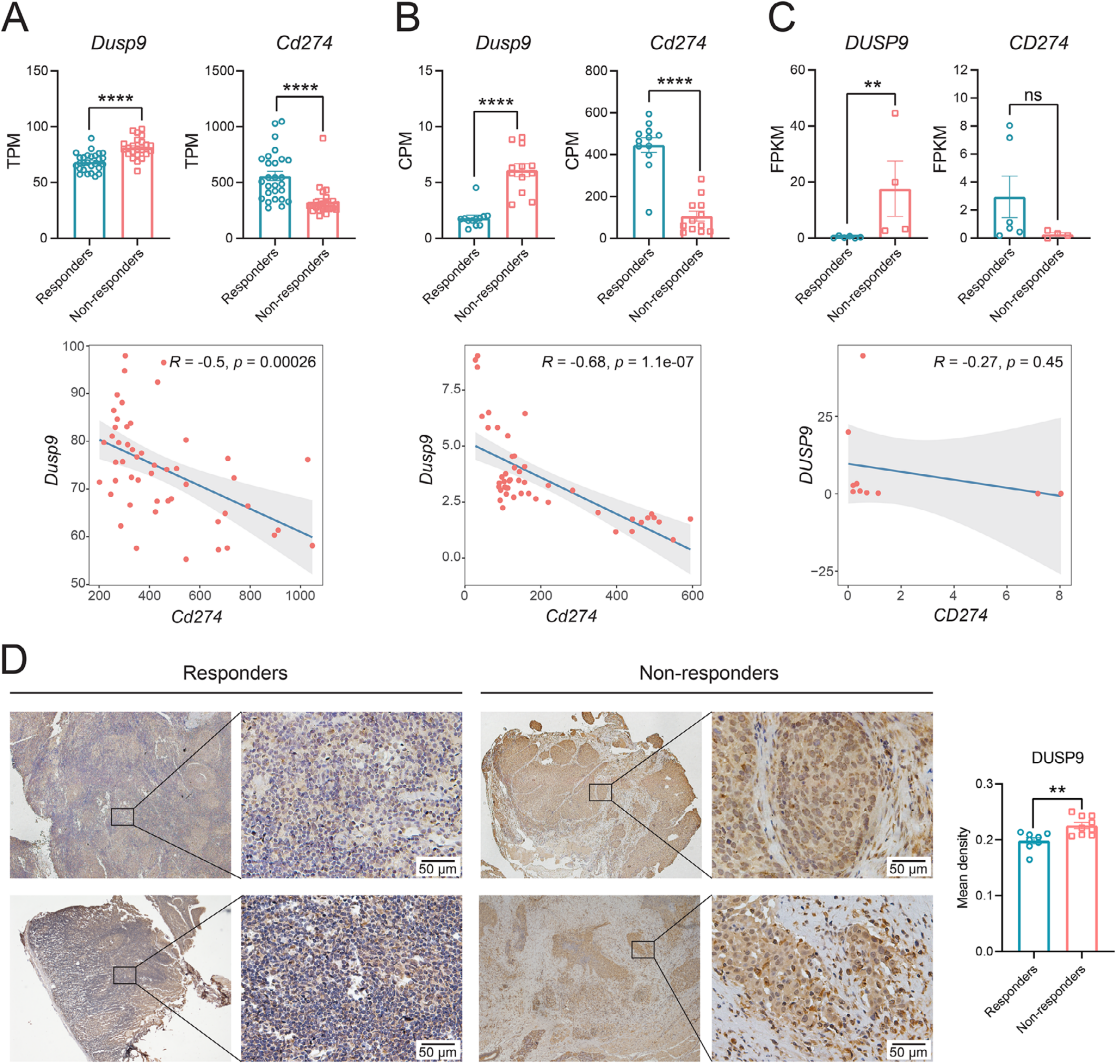
DUSP9 may serve as a clinical predictor of ICB therapy efficacy. A–C) The mRNA expression of DUSP9 and PD‐L1 between responders and non‐responders in various tumors was investigated: (A) melanoma treated with anti‐PD‐L1 therapy (reanalysis of the GSE172320 dataset); (B) mesothelioma treated with anti‐CTLA4 and anti‐PD‐L1 therapy (reanalysis of the GSE117358 dataset); (C) HCC treated with anti‐PD‐L1 therapy (reanalysis of the GSE279750 dataset). The scatter plots show the correlation between the mRNA expression levels of DUSP9 and PD‐L1 according to each dataset. D) Immunohistochemistry results show the expression of DUSP9 in responders and non‐responders to anti‐PD‐1/PD‐L1 therapy among clinical HNSC patients. Data are shown as mean ± SEM; *p* values were determined by Student's t‐test (A, B, D) or Mann‐Whitney test (C); ns, not significant; ^**^, *p* < 0.01; ^****^, *p* < 0.0001.

While investigating the clinical significance of PD‐L1 and DUSP9 in patients receiving immunotherapy, we found that those with high PD‐L1 expression had better survival outcomes, while those with high DUSP9 expression had worse outcomes (Figure 1L). We searched the GEO database for public transcriptome data on immunotherapy responsiveness. One study that characterized PD‐L1 blockade in a melanoma model showed that DUSP9 expression was lower in the responder group than in the non‐responder group, whereas PD‐L1 expression was higher in the responder group (**Figure** **7**A). Another study on mesothelioma treated with CTLA‐4 and PD‐L1 immunotherapy revealed a comparable pattern (Figure 7B). In a different clinical trial of anti‐PD‐L1 therapy for unresectable HCC, DUSP9 and PD‐L1 expression remained inversely correlated with patient response, and a significant increase in DUSP9 expression was observed in the non‐responder group (Figure 7C). The correlation analysis of DUSP9 and PD‐L1 expression in the aforementioned bulk datasets revealed a negative correlation (Figure 7A–C, lower panels).

We collected clinical samples from HNSC patients and divided them based on their response to anti‐PD‐1/PD‐L1 treatment. We found that DUSP9 expression was higher in the non‐responding patient population (Figure 7D). These results suggest that DUSP9 may be a predictor of anti‐PD‐1 therapy efficacy.

## 3 Discussion

In this study, we developed a mutual exclusion analysis system for PD‐L1 expression, which considers mixed cell phenotypes. Mutual exclusion (ME) differs from negative correlation. Mutually exclusive gene pairs can only have one highly expressed gene or both genes with low expression.^[^ ^16^ ^]^ In contrast, negatively correlated gene pairs may be highly expressed simultaneously. In cell‐type‐specific analyses, some mutually exclusive genes are generally not expressed in the cell line. This results in low plasticity, leading to their omission in the screening process. This study employs a method that increases PD‐L1 plasticity and improves the ability to identify ME genes. For example, of the 57 ME genes obtained under strict filtering conditions in this study, 37 could not be obtained through previous cell‐specific screening.^[^ ^16^ ^]^ Of the remaining 20 genes, 17 can be screened out through a single cell line. The remaining three genes—APOC1 (apolipoprotein C1), DUSP9, and SERPINF1 (serpin family F member 1)—can be obtained through two cell lines.^[^ ^16^ ^]^ Therefore, these results suggest that combining transcriptome data from various cell sources to increase PD‐L1 expression gene plasticity could improve ME analysis efficiency.

Our previous studies^[^ ^16^, ^23^, ^24^ ^]^ and this study revealed that ME may imply negative regulation. Among the 57 genes examined, FGFR3,^[^ ^26^ ^]^ SYK (spleen associated tyrosine kinase),^[^ ^44^ ^]^ and APOC1,^[^ ^45^ ^]^ were previously reported to negatively regulate PD‐L1 expression. This study identified DUSP9, CORO1A, FGFR4, and KCNH2 as novel PD‐L1 regulators. Relaxing the filter conditions may identify more potential negative regulators. For example, NDRG2 (NDRG family member 2) was previously reported to negatively regulate PD‐L1 expression.^[^ ^46^ ^]^ The delta value and cosine similarity to CD274 are −20.62 (*p* = 1.02E‐15) and 0.09963, respectively (Table S1, Supporting Information). Additionally, METTL7B negatively regulates PD‐L1 expression.^[^ ^16^, ^47^ ^]^ The delta value and cosine similarity of METTL7B to CD274 are −14.4 (*p* = 2.02E‐07) and 0.07779, respectively (Table S1, Supporting Information).

This study used PD‐L1 as the source gene to divide the samples into PD‐L1^high^ and PD‐L1^low/−^ groups for virtual sorting. However, other genes, such as ESR1 (estrogen receptor 1) and NR1H4 (nuclear receptor subfamily 1 group H member 4), can also be used to categorize samples. In this case, PD‐L1 could be identified as an ME molecule (Table S1, Supporting Information). ESR1^[^ ^48^ ^]^ and NR1H4^[^ ^49^ ^]^ have been identified as negative regulators of PD‐L1 expression. Using the same filtering criteria (cosine similarity ≤ 0.05, δ ≤‐15, and adjusted *p* value ≤ 0.01), an additional 185 genes were identified as PD‐L1 ME candidates when all protein‐coding genes served as the source genes for virtual sorting (Table S1, Supporting Information). This is an important and valuable resource for identifying PD‐L1 regulators.

Therefore, we believe that screening negative regulators of PD‐L1 expression through ME analysis is efficient and cost‐effective, especially when supported by omics big data, which makes the method highly reliable. However, the experimental methods for systematically screening these genes, such as genetic methods, are time‐consuming and costly. For example, several studies have employed CRISPR/Cas9 technology to screen PD‐L1 regulatory genes.^[^ ^50^, ^51^, ^52^ ^]^ However, genetic screening is better suited to positive regulation. This is mainly because once the cells used for screening have extremely low or no expression of mutually exclusive genes, knocking out these genes may not have a biological effect. Conversely, knocking out a key gene that positively regulates PD‐L1 expression easily shows a biological effect. Consequently, multiple studies have used CRISPR/Cas9 to screen out JAK1, JAK2, STAT3, and other key positive regulatory molecules in signaling pathways that control PD‐L1 expression.^[^ ^53^, ^54^, ^55^, ^56^ ^]^

However, it should be noted that we initially explored mutual exclusion to predict immune cell phenotypes based on omics big data and the rules by which they change during phenotypic conversion.^[^ ^17^, ^18^, ^23^ ^]^ This principle also applies to non‐immune cells in this study. For example, ME analysis and experimental verification demonstrate that PD‐L1^high^ tumor cells exhibit low or no DUSP9 expression (DUSP9^low/−^), while DUSP9^high^ tumor cells exhibit low or no PD‐L1 expression (PD‐L1^low/−^). Similar findings were observed for METTL7B, a negative regulator of PD‐L1 that exhibits mutually exclusive expression with PD‐L1.^[^ ^16^ ^]^ Significant PD‐L1 upregulation depends on low or absent METTL7B expression. When METTL7B is not downregulated, PD‐L1 cannot be significantly upregulated.^[^ ^16^ ^]^ Therefore, mutual exclusion analysis can predict changes in the molecular phenotype of cells, especially for mutually exclusive genes with causal connections. This intrinsic relationship between genes is called the ‘internal phenotype’, and the process by which it is regulated is called ‘internal phenotypic regulation’.^[^ ^23^ ^]^

This study proposes new methods for predicting immunotherapy responsiveness and developing combination therapy strategies, based on an analysis of PD‐L1's mutually exclusive genes. Previous studies have shown that patients with high PD‐L1 expression benefit more from anti‐PD‐1/PD‐L1 treatment ^[^ ^11^, ^12^ ^]^ and that increased PD‐L1 expression during treatment is more closely associated with a positive response.^[^ ^14^ ^]^ PD‐L1 expression is a logical biomarker for predicting a positive response to anti‐PD‐1/PD‐L1 immunotherapies; however, its predictive value is currently controversial and challenging due to its dynamic expression level and limited detection methods.^[^ ^14^ ^]^ Therefore, screening for high expression of PD‐L1's ME genes is expected to identify patients who will not respond to ICB therapy. However, this study has limited data on patient and tumor types. Further exploration is needed to predict therapeutic responses through the combination of multiple ME genes.

This study demonstrates that DUSP9 negatively regulated PD‐L1 transcription through dephosphorylation of STAT3. DUSP9 exhibits broad dephosphorylation specificity for many substrates, including JNK, p38, ERK, mTOR and ASK1.^[^ ^57^, ^58^, ^59^, ^60^ ^]^ STAT3 has been reported to be phosphorylated by various kinases, such as JAK and Src,^[^ ^61^, ^62^ ^]^ and dephosphorylated by protein tyrosine phosphatases (PTPs), including phosphatases from the dual‐specificity phosphatase family (DUSP2, DUSP3 and DUSP22).^[^ ^63^, ^64^, ^65^ ^]^ STAT3 has been reported to act on the PD‐L1 promoter to increase its expression.^[^ ^29^, ^30^, ^66^ ^]^ However, the relationship between DUSP9 and STAT3 remains unclear. This study identified p‐STAT3 as a novel DUSP9 substrate. We examined STAT3 phosphorylation at two sites and found that DUSP9 knockdown increased STAT3 phosphorylation and nuclear translocation. The loss of STAT3 prevents the negative regulatory effect of DUSP9 on PD‐L1, indicating that DUSP9's negative regulation of PD‐L1 depends on its dephosphorylation of STAT3.

Interfering with DUSP9 expression could increase PD‐L1 expression, particularly in cold tumors. Targeting DUSP9 in combination with a PD‐1 antibody could improve therapeutic sensitivity in mouse models of colorectal (MC38) and Lewis lung (LLC) carcinomas, particularly the latter, which is a cold tumor model. Tumors that lack PD‐1 or PD‐L1 expression are called as ‘target‐missing’.^[^ ^43^ ^]^ Target‐missing resistance occurs in 60–85% of solid tumors, representing a major reason why only a small fraction of patients respond to anti‐PD‐1/PD‐L1 therapy.^[^ ^43^ ^]^ Therefore, interfering with PD‐L1 ME molecules, such as by targeting DUSP9, could help overcome ‘target‐missing’ resistance and provide new directions for exploring tumor combination immunotherapy.

The development of specific DUSP9 inhibitors has the potential to enhance the efficacy of cancer immunotherapy as an adjuvant therapy. Additionally, DUSP9's specific expression pattern, which is high in the placenta and tumors and low in normal tissues, is similar to that of carcinoembryonic antigens, making it a promising therapeutic target. However, phosphatases are often described as undruggable by traditional strategies, so developing DUSP9 inhibitors still faces challenges. Recently, innovative pharmacological approaches targeting phosphatases have emerged. Allosteric inhibitors that target DUSP family members DUSP1 and DUSP6 can bind near the catalytic site of phosphatases, hindering the allosteric changes required for substrate binding.^[^ ^67^ ^]^ Therefore, the structural basis of the interaction between DUSP9 and STAT3 requires further exploration.

The inhibitory role of DUSP9 knockdown in tumor growth was verified through in vitro colony formation and in vivo tumor formation in nude mice, which is consistent with previous research.^[^ ^37^ ^]^ This study demonstrates that DUSP9 plays a dual role in inhibiting tumor growth and negatively regulating PD‐L1 expression. This indicates the complex interplay between cell‐intrinsic (pro‐proliferative) and cell‐extrinsic (immunomodulatory) functions within an intact immune system. Therefore, when developing small‐molecule inhibitors aimed at targeting tumor cell proliferation, attention should be paid to their potential impact on the immune microenvironment. In this context, combining small molecule inhibitors with PD‐1/PD‐L1 antibodies may achieve better therapeutic effects, providing a new approach to combining targeted and immunotherapies in clinical settings.

Of the 57 genes, targeting some of them, such as SYK,^[^ ^44^ ^]^ SERPINF1,^[^ ^68^ ^]^ APOC1,^[^ ^45^ ^]^ and CORO1A,^[^ ^69^ ^]^ objectively increased the response to ICB immunotherapy. For example, PD‐L1 expression increased in tumor cells and macrophages in a mouse neuroblastoma tumor model following treatment with the SYK inhibitor R788. R788 also made neuroblastoma tumors more susceptible to ICB immunotherapy.^[^ ^44^ ^]^ In the MC38 model, *Serpinf1* overexpression induced resistance to anti‐PD‐1 antibodies. Conversely, *Serpinf1* knockdown sensitized this model as well as the primarily resistant MBT2 murine bladder cancer model.^[^ ^68^ ^]^ APOC1 was found to be negatively correlated with PD‐L1 expression in human hepatocellular carcinoma (HCC) samples.^[^ ^45^ ^]^ PD‐L1 expression increased in *Apoc1* ^−/−^ mice. Furthermore, APOC1 deficiency increased sensitivity to anti‐PD‐1 therapy in the H22 murine hepatocellular carcinoma model.^[^ ^45^ ^]^ Additionally, *Coro1a* knockout reversed anti‐PD‐L1 therapy resistance.^[^ ^69^ ^]^ Therefore, targeting ME genes in combination with anti‐PD‐1/PD‐L1 antibodies has shown increased anti‐tumor efficiency. This suggests that the mutual exclusion (ME)‐interference (MEi) strategy provides a new approach to combining ICB immunotherapy and sensitizing the therapeutic response of cold tumors.

However, this study has several limitations. First, we screened IFN‐γ‐downregulated genes in the A549 model, which may have overlooked other genes that are mutually exclusive and not expressed in A549 cells. For example, GPC3 is barely expressed in A549 cells yet is included in the list of 57 genes. However, GPC3 is highly expressed in liver cancer.^[^ ^16^ ^]^ In patients with hepatocellular carcinoma, combination therapy with atezolizumab (anti‐PD‐L1) and bevacizumab (anti‐VEGF) showed reduced clinical benefit associated with GPC3 expression. Conversely, patients with high CD274 expression showed improved progression‐free survival (PFS) and overall survival (OS).^[^ ^70^ ^]^ A larger OS treatment benefit was observed in the population with low GPC3 expression.^[^ ^70^ ^]^ Since GPC3 expression is mutually exclusive with PD‐L1 expression (Table S1, Supporting Information), these clinical results support our study's hypothesis. Therefore, mutually exclusive genes may inhibit PD‐L1 in different tumor types. Second, further comprehensive clinical cohort data are needed to fully determine the predictive role of the DUSP9 combined with PD‐L1 in tumor immunotherapy. Third, this study used mixed cell phenotypes to increase gene plasticity and improve screening sensitivity for the PD‐L1 ME molecule. However, it cannot be ruled out that some ME molecules with low plasticity were missed due to the limited sample size and conditions.

In conclusion, we have established a mutually exclusive screening system for PD‐L1 expression and demonstrated that DUSP9 negatively regulates PD‐L1 expression by dephosphorylating STAT3. Knocking down of DUSP9 enhances sensitivity to PD‐1 antibody therapy. Thus, DUSP9 is expected to be a new target molecule for tumor immunotherapy when used in combination with anti‐PD‐1/PD‐L1 antibodies, as well as a new clinical biomarker for predicting responsiveness to ICB therapy.

## 4 Experimental Section

### RNA Sequencing Data Analysis

Information about samples related to human primary cells or cell lines was retrieved from the Gene Expression Omnibus (GEO) database (https://www.ncbi.nlm.nih.gov/geo/). Next, the publicly available sequencing data from 600 randomly selected samples were downloaded from the Sequence Read Archive (SRA) database (https://www.ncbi.nlm.nih.gov/sra) (Table S3, Supporting Information). Quality control, read mapping to the human genome (GRCh38), and expression quantification (including calculating transcripts per million [TPM] at the gene level) have been described previously.^[^ ^16^ ^]^ Public scRNA‐Seq datasets for human tumor samples were sourced from GSE123139 (melanoma),^[^ ^71^ ^]^ GSE125449 (HCC),^[^ ^72^ ^]^ and GSE131907 (LUAD).^[^ ^73^ ^]^ The data were analyzed using the R package Seurat (v4.1.1) (https://satijalab.org/seurat/), following a standardized pipeline as previously described.^[^ ^18^ ^]^ Cell types were annotated according to the original paper or marker genes, and the results were visualized using R functions of the Seurat package.

### Gene Plasticity Analysis

As previously described,^[^ ^16^ ^]^ the percentile rank scores for each gene in a sample were first calculated based on the TPM values using the following formula: P = n/N × 100, where P is the percentile rank score; N, the number of non‐zero TPM values; and n, the ordinal rank of a gene when all genes’ TPM values were in ascending order in each assay. When a gene's TPM value equals zero, its percentile rank score was also set to zero. Therefore, percentile rank scores range from zero to 100, corresponding to low to high expression. The average rank score (ARS) of a gene was the mean of its percentile rank scores across all samples and represents its average expression level across various conditions. In this study, the gene plasticity (GP) score was defined as the difference between the 5th and 95th percentile rank scores across different samples (i.e., Delta_95/5_). This range of quantiles allowed at least 10% of the total samples to support expression plasticity while avoiding potential outliers due to the experimental design. Thus, the gene plasticity observed in this study reflects changes in gene expression ranking.

### Virtual Sorting Based on Changes in Gene Plasticity

First, the samples were divided into two groups using the k‐means clustering method (k = 2) based on the percentile rank scores of PD‐L1 across samples: PD‐L1 high (PD‐L1^high^) and PD‐L1 low (PD‐L1^low/−^). The PD‐L1^high^ group exhibited relatively high PD‐L1 expression, whereas the PD‐L1^low/−^ group displayed low or absent PD‐L1 expression. For each group, the ARS of each gene was calculated. Then, the differences (δ_ARS_) between the ARSs of the two groups were ranked in order of decreasing magnitude. The top and bottom genes of the ranked list were identified as positively (co‐existing) or anti‐ (mutually exclusive) correlated gene sets based on a δ_ARS_ cutoff. For this study, the cutoffs were set at greater than or equal to 15 and less than or equal to −15, respectively, for correlated and anti‐correlated gene sets. The Wilcoxon rank sum test was used to analyze statistical significance. *p*‐values were corrected for multiple testing using the Benjamini‐Hochberg method. An adjusted *p* value of less than or equal to 0.01 indicates a significant difference.

### Cosine Similarity

For cosine similarity (cos), the cosine values were calculated using the following formula:

(1) $$\cos\left(\theta \right)=\frac{\underset{i=1}{\sum^{n}}\left(x_{i}\cdot y_{i}\right)}{\sqrt{\underset{i=1}{\sum^{n}}{x_{i}}^{2}}\sqrt{\underset{i=1}{\sum^{n}}{y_{i}}^{2}}}$$

In this formula, (x_1_, x_2_, ⋯, x_n_) and (y_1_, y_2_, ⋯, y_n_) represent the sets of transcripts per million (TPM) values for two genes in the same sample (n = 600). The formula uses the angle between vectors X and Y to represent θ, and the cosine similarity varies from 0 to 1. When two vectors were oriented the same way, the angle between them was 0°, and the cosine similarity was 1. However, when two vectors form a 90° angle, the cosine similarity was 0.

### Cell Culture

The following cell lines were purchased from the American Type Culture Collection (ATCC): the human lung carcinoma cell line A549 (Cat#CCL‐185, RRID: CVCL_0023); the cervical carcinoma cell line HeLa (Cat#CCL‐2, RRID: CVCL_0030); the embryonic kidney cell line HEK293T (Cat#CRL‐3216, RRID: CVCL_0063); and the mouse liver carcinoma cell line Hepa1‐6 (Cat#CRL‐1830, RRID: CVCL_0327). The FaDu human oropharyngeal carcinoma cell line (Cat#CL‐0083, RRID: CVCL_1218), Huh7 human liver carcinoma cell line (Cat#CL‐0120, RRID: CVCL_0336), and MC38 mouse colon carcinoma cell line (Cat#CL‐0972, RRID: CVCL_B288) were purchased from Procell (Wuhan, China). The LLC mouse lung carcinoma cell line (RRID: CVCL_4358) was kindly provided by Prof. Chuanhui Han (Peking University International Cancer Institute). The FaDu cell line was cultured in minimum essential medium (MEM, Cat#SH30024, Cytiva). The other cell lines were cultured in Dulbecco's modified Eagle medium (DMEM, Cat#SH30022, Cytiva). All media were supplemented with 10% fetal bovine serum (FBS, Cat#HK‐CH500, HUANKE) and 1% penicillin‐streptomycin (Cat#P1400, Solarbio). All cells were verified as mycoplasma‐free.

### Patient Samples

Human HNSC tissue samples were obtained from Beijing Tongren Hospital. This study was approved by the Ethics Committee of Beijing Tongren Hospital (item number TREC2023‐KY009.A1). All participants involved in this study signed informed consent forms.

### Animal Experiments

Six‐week‐old male BALB/c nude and C57BL/6 mice were obtained from the Department of Laboratory Animal Science at Peking University Health Science Center. All procedures involving the mice were approved by the Institutional Animal Care and Use Committee of the Peking University Health Science Center (protocol number 2022135). The mice were housed under specific pathogen‐free conditions and had ad libitum access to food and water. Each mouse was considered an experimental unit within the studies. The sample size for each group was determined based on previous literature and research. Animals were randomized using a computerized random order generator and treated and measured at random. No criteria were established for the inclusion or exclusion of animals during the experiment.

### Tumor Models and Treatments

For the immunodeficient mouse model, Hepa1−6 (3 × 10^6^ cells), MC38 (3 × 10^6^ cells), and LLC (2 × 10^6^ cells) cells from the shNC and sh*Dusp9* groups were injected subcutaneously into the BALB/c nude mice. For the immunocompetent mouse model, MC38 (5 × 10^6^ cells) and LLC (3 × 10^6^ cells) cells from the shNC and sh*Dusp9* groups, wild‐type MC38 (3 × 10^6^ cells) and LLC (2 × 10^6^ cells) cells were injected subcutaneously into the C57BL/6 mice, and MC38 cells were injected with 30% Matrigel (Cat#354234, Corning). For the anti‐PD‐1 treatment, PD‐1 monoclonal antibody (Cat#BE0146, BioXcell) and rat IgG2a isotype (Cat#BE0089, BioXcell) were injected intraperitoneally. To transfect *Dusp9‐*targeting siRNA in vivo, the GNT siRNA trans 002 transfection reagent (Cat#0010102010, Genable [Beijing] Biotechnology) and *siDusp9* were mixed according to the instructions and injected intratumorally. Mouse tumor volumes were measured every three days. Tumor volumes were calculated as 1/2 × length × width^2^. A tumor volume of 1000 mm^3^ was used as the humane endpoint. Two investigators performed treatment and measurement in a blinded fashion.

### Reagents and Antibodies

The STAT3 inhibitor S3I‐201 (NSC74859, Cat#S1155) was purchased from Selleck. Recombinant human IFN‐γ (Cat#300‐02) and recombinant murine IFN‐γ (Cat#315‐05) were purchased from PeproTech. Human recombinant DUSP9 (Cat#AG5881) was purchased from Proteintech. Calf intestine alkaline phosphatase (CIAP, Cat#10321ES80) was purchased from Yeasen. Live/dead dye 7‐aminoactinomycin D (7‐AAD, Cat#420404) was purchased from BioLegend, and the fixable viability dye eFluor 506 (Cat#65‐0866‐14) was purchased from eBioscience.

The primary antibodies used for Western blot, immunohistochemistry (IHC), immunofluorescence (IF), immunoprecipitation (IP), and Co‐immunoprecipitation (Co‐IP) include the following: anti‐human PD‐L1 (Cat#13684, Cell Signaling Technology), anti‐mouse PD‐L1 (Cat#ab213480, Abcam), anti‐human/mouse PD‐L1 (Cat#66248‐1‐Ig, Proteintech), anti‐DUSP9 (Cat#FNab02571, FineTest), anti‐p‐STAT3‐Y705 (Cat#9145, Cell Signaling Technology), anti‐p‐STAT3‐S727 (Cat#9134, Cell Signaling Technology), anti‐STAT3 (Cat#12640, Cell Signaling Technology), anti‐CD8 (Cat#sc‐1177, Santa Cruz), anti‐GZMB (Cat#13588‐1‐AP, Proteintech) and anti‐β‐actin (Cat#TA‐09, ZSGB‐Bio). Secondary antibodies used for Western blot and Co‐IP include HRP‐conjugated anti‐mouse IgG (Cat#7076S, Cell Signaling Technology), HRP‐conjugated anti‐rabbit IgG (Cat#7074S, Cell Signaling Technology), and HRP‐conjugated anti‐rabbit IgG for IP (Cat#RA1008, Vazyme). The secondary antibodies used for immunofluorescence (IF) include FITC‐conjugated goat anti‐mouse IgG (Cat#SA00003‐1, Proteintech) and TRITC‐conjugated goat anti‐rabbit IgG (Cat#SA00007‐2, Proteintech). The fluorescent antibodies used in flow cytometry were all purchased from BioLegend and included APC‐anti‐human‐PD‐L1 (Cat#329708), APC‐anti‐mouse‐PD‐L1 (Cat#124312), AF700‐anti‐mouse‐CD45 (Cat#103128), FITC‐anti‐mouse‐CD3 (Cat#100204), PE‐Cy7‐anti‐mouse‐CD4 (Cat#100528), BV605‐anti‐mouse‐CD8 (Cat#100744), PerCP‐Cy5.5‐anti‐human/mouse‐GZMB (Cat#372211), PE‐anti‐mouse‐IFN‐γ (Cat#505808), and APC‐anti‐mouse‐TNF‐α (Cat#506308).

### Plasmids and RNA Interference

The mammalian expression vector for DUSP9 overexpression was constructed using the pcDNA3.1‐Myc‐His vector. The STAT3 overexpression vector was kindly provided by Prof. Yanhui Yin.^[^ ^74^ ^]^ Lentiviral vector plasmids carrying short hairpin RNAs (shRNAs) were constructed using the pLKO.1‐copGFP‐2A‐PURO vector from Tsingke. Small interfering RNAs (siRNAs) were purchased from Tsingke. The targeting sequences were listed in Table S4 (Supporting Information). All the plasmids were transfected into cells using the following transfection reagents: Lipofectamine 3000 (Cat#L3000015, ThermoFisher) or jetPRIME (Cat#101000046, Polyplus) for plasmids, and Lipofectamine RNAiMAX (Cat#13778‐150, ThermoFisher) for siRNAs.

### Generation of DUSP9 Stable Knockdown Tumor Cell Lines

Lentiviruses were packaged in HEK293T cells using the jetPRIME transfection reagent (Cat#101000046, Polyplus). The plasmid ratio was pLKO.1:psPAX2:pCMV‐VSV‐G = 3:2:1. The supernatant was collected 48 h after transfection. Tumor cells were plated in six‐well plates and cultured with a mixture of one milliliter of complete medium and one milliliter of the virus supernatant for 24 h. Polybrene (Cat#H8761, Solarbio) was added to the mixture at a final concentration of 10 µg mL^−1^. After 48 h of infection, puromycin (Cat#ST551, Beyotime) selection was performed for one week at the lowest killing concentration: 0.5 µg mL^−1^ for Hepa1−6, 2 µg mL^−1^ for MC38, and 1 µg mL^−1^ for LLC.

### Colony Formation Assay

Hepa1‐6, MC38, and LLC tumor cell lines from the shNC and sh*Dusp9* groups were seeded at a density of 1 × 10^3^ cells per well in six‐well plates. After two weeks, the cells were fixed with 4% paraformaldehyde and stained with crystal violet. The number of colonies was quantified using ImageJ (NIH).

### Real‐Time Quantitative PCR (RT‐qPCR)

Total RNA was extracted using a total RNA extraction reagent (Cat#R401‐01‐AA, Vazyme), and reverse transcription reactions were performed using a HiScript III All‐In‐One RT SuperMix (Cat#R333‐01, Vazyme). The primer sequences used in RT‐qPCR are listed in Table S5 (Supporting Information).

### Western Blot

The cells were washed with PBS and lysed with RIPA lysis buffer (Cat#P0013C, Beyotime), which was supplemented with protease (Cat#04693132001, Roche) and phosphatase inhibitors (Cat#04906845001, Roche). The lysates were then centrifuged to remove debris, after which the protein concentrations were measured using the BCA reagent (Cat#23227, ThermoFisher). The precipitates were boiled in 1× SDS loading buffer for 10 min at 99°C. The proteins were separated by SDS‐PAGE gels and transferred onto nitrocellulose membranes (Cat#10600002, Cytiva). The membranes were then immunoblotted with different antibodies and detected using the ImageQuant LAS‐500 system (Cytiva). Signals were quantified using ImageJ (NIH).

### Immunoprecipitation‐Mass Spectrometry (IP‐MS) and Co‐Immunoprecipitation (Co‐IP)

Huh7 cells were stimulated with human IFN‐γ (50 ng mL^−1^) for 4 h, then washed with PBS and lysed with lysis buffer (Cat#P0013, Beyotime), which was supplemented with protease (Cat#04693132001, Roche) and phosphatase (Cat#04906845001, Roche) inhibitors. The lysates were then centrifuged to remove debris. The resulting supernatants were pre‐incubated with IP antibodies for 2 h, after which protein G beads (Cat#17061801, Cytiva) were added and the mixture was incubated overnight at 4°C. After incubation, the sepharose beads were centrifuged and washed five times with pre‐cold PBS. The precipitates were boiled in 1× SDS loading buffer for 10 min at 99°C. The proteins were then separated by SDS‐PAGE gels and analyzed by mass spectrometry (IP‐MS) or Western blot (Co‐IP).

### In Vitro Dephosphorylation Assay

HeLa cells were transfected with HA‐STAT3 for 48 h and then stimulated with human IFN‐γ (50 ng mL^−1^) for 4 h. The cells were then processed as described in the IP procedure. The antibody used for the IP was anti‐HA (Cat#51064‐2‐AP, Proteintech). The HA‐STAT3‐bound beads were washed and divided into each sample tube. Each sample was mixed with DUSP9 (5 µg) or CIAP (30 U) in 100 µL buffer solution containing 50 mmol L^−1^ imidazole (pH7.5) and 5 mmol L^−1^ dithiothreitol. The samples were then incubated at 37°C for 1 h, after which the reaction was stopped by boiling the samples in SDS loading buffer (1×) for 10 min at 99°C. The proteins were then separated by SDS‐PAGE gels and analyzed by Western blot.

### Immunohistochemistry

Tissue specimens from HNSC patients were embedded in paraffin and sectioned into 4 µm slices. The sections were incubated with primary antibodies at 4°C overnight, followed by incubation with an HRP‐conjugated secondary antibody (Cat#GK600705, GeneTech) at room temperature. The sections were developed with diaminobenzidine (DAB) and counterstained with hematoxylin. Images were captured using a BZ‐X800 microscope (Keyence), and the mean densities were quantified using ImageJ (NIH).

### Immunofluorescence

Tissue specimens from HNSC patients were embedded in paraffin and sectioned into 4 µm slices. The sections were incubated with primary antibodies at 4°C overnight, followed by incubation with fluorescence‐conjugated secondary antibodies at room temperature. The sections were then incubated with DAPI (Cat#C0060, Solarbio). Images were captured using confocal microscopy (Leica).

Tissue specimens from mice were embedded in an optimal cutting temperature compound (OCT, Cat#4583, Sakura) and sectioned into 10 µm sections. After being treated with 0.1% Triton‐X100 (Cat#0694, Amresco), the sections were incubated with primary antibodies at 4°C overnight. The subsequent steps were performed similarly to those for the paraffin sections.

### Flow Cytometry

A single‐cell suspension was obtained from a mouse tumor by rapid and gentle dissection, grinding, and filtration. Immune cells and tumor cells were isolated using 70% and 40% Percoll (Cat#17089101, Cytiva). The cells were stained with fluorescence‐conjugated antibodies targeting membrane molecules at 4°C. After being treated with IC fixation buffer (Cat#00‐8222‐49, Invitrogen) and permeabilization buffer (Cat#00‐8333‐56, Invitrogen), the intracellular molecules were stained. The cells were acquired by flow cytometry using a FACS Canto (BD). The data were analyzed using FlowJo (BD).

### Co‐Culture Assay of T Cells and Tumor Cells

Lymph nodes were collected from six‐week‐old wild‐type C57BL/6 mice to acquire T cells. The T cells were activated with 2 µg mL^−1^ anti‐CD3 (Cat#100340, Biolegend) and 1 µg mL^−1^ anti‐CD28 (Cat#102116, Biolegend) for 24 h. *Dusp9* stable knockdown (sh*Dusp9*) and negative control (shNC) Hepa1‐6 cells were seeded in 96‐well plates and allowed to adhere overnight. Then, the activated T cells were transferred to incubate with tumor cells at a ratio of 5:1 for 48 h. To block PD‐1/PD‐L1, 10 µg mL^−1^ of PD‐1 monoclonal antibody (Cat#BE0146, BioXcell) and rat IgG2a isotype (Cat#BE0089, BioXcell) were added at the beginning of the co‐culture assay.

### Statistical Analysis

All statistical analyses and graphs in this paper were performed using GraphPad Prism 8.0. Data were presented as mean ± standard error of the mean (SEM) for the biological replicates. The sample size and statistical analysis methods were indicated in the figure legends. Differences between two groups were analyzed using a two‐tailed Student's t‐test or a Mann‐Whitney test, while differences between multiple groups were analyzed using a one‐way or two‐way ANOVA test. A *p*‐value of less than 0.05 was considered statistically significant. P values are reported as follows: ^*^ for *p* < 0.05, ^**^ for *p* < 0.01, ^***^ for *p* < 0.001, ^****^ for *p* < 0.0001, or ns for not significant.

## Conflict of Interest

The authors declare no conflict of interest.

## Author Contributions

P.W. conceived the study. P.W. and W.H. supervised experiments and revised the manuscript. W.G. collected the clinical samples and revised the manuscript. Y.H. performed experiments and drafted the manuscript. P.W. and Y.H. performed bioinformatic analysis. L.T., Z.K., and D.H. participated in data analysis. T.L., G.Y., and Y.C. assisted with data analysis and offered suggestions. All authors contributed to the article and approved the submitted version.

## Supporting information



Supporting Information

Supporting Information

Supporting Information

Supporting Information

Supporting Information

Supporting Information

## Data Availability

The data that support the findings of this study are available from the corresponding author upon reasonable request.
