# Supplementary material for: Mutual Exclusion Analysis Shows that DUSP9 Negatively Regulates PD‐L1 Expression and Acts as a Target to Enhance Anti‐PD‐1 Efficacy
Source: Adv Sci (Weinh). 2025 Dec 17;13(12):e14347. doi: 10.1002/advs.202514347 (PMC12948242; doi:10.1002/advs.202514347)
Supplement: Supplementary file 1 — Supporting Information [file ADVS-13-e14347-s006.pdf]

Supplementary Figures

Title: Mutual Exclusion Analysis Shows that DUSP9 Negatively Regulates PD-L1 Expression and Acts as a Target to Enhance Anti-PD-1 Efficacy

Authors: *Yuzhe Hu, Ling Tang, Zheng Kuang, Danyi Huang, Ting Li, Gaofei Yin, Yingyu Chen, Wei Guo,* Wenling Han,* and Pingzhang Wang**

Affiliations:

Y. Hu, L. Tang, Z. Kuang, D. Huang, T. Li, Y. Chen, W. Han, P. Wang
Department of Immunology, NHC Key Laboratory of Medical Immunology (Peking University), Medicine Innovation Center for Fundamental Research on Major Immunology-related Diseases, School of Basic Medical Sciences, Peking University Health Science Center, Beijing, 100191, China.
E-mail: W. Han (hanwl@bjmu.edu.cn), P. Wang (wangpzh@bjmu.edu.cn).

Y. Hu, L. Tang, Z. Kuang, D. Huang, T. Li, Y. Chen, W. Han, P. Wang
Peking University Center for Human Disease Genomics, No. 38 Xueyuan Road, Beijing, 100191, China.

G. Yin, W. Guo
Department of Otorhinolaryngology Head and Neck Surgery, Beijing Tongren Hospital, Capital Medical University, Key Laboratory of Otolaryngology Head and Neck Surgery (Capital Medical University), Ministry of Education, Beijing, 100730, China.
E-mail: W. Guo (entguowei@sina.com).

Funding: National Natural Science Foundation of China (No. 32270990), Beijing Municipal Natural Science Foundation (No.7232095).

Keywords: PD-L1, DUSP9, immune checkpoint blockade (ICB), combination therapy, mutual exclusion interference (MEi) strategy, STAT3

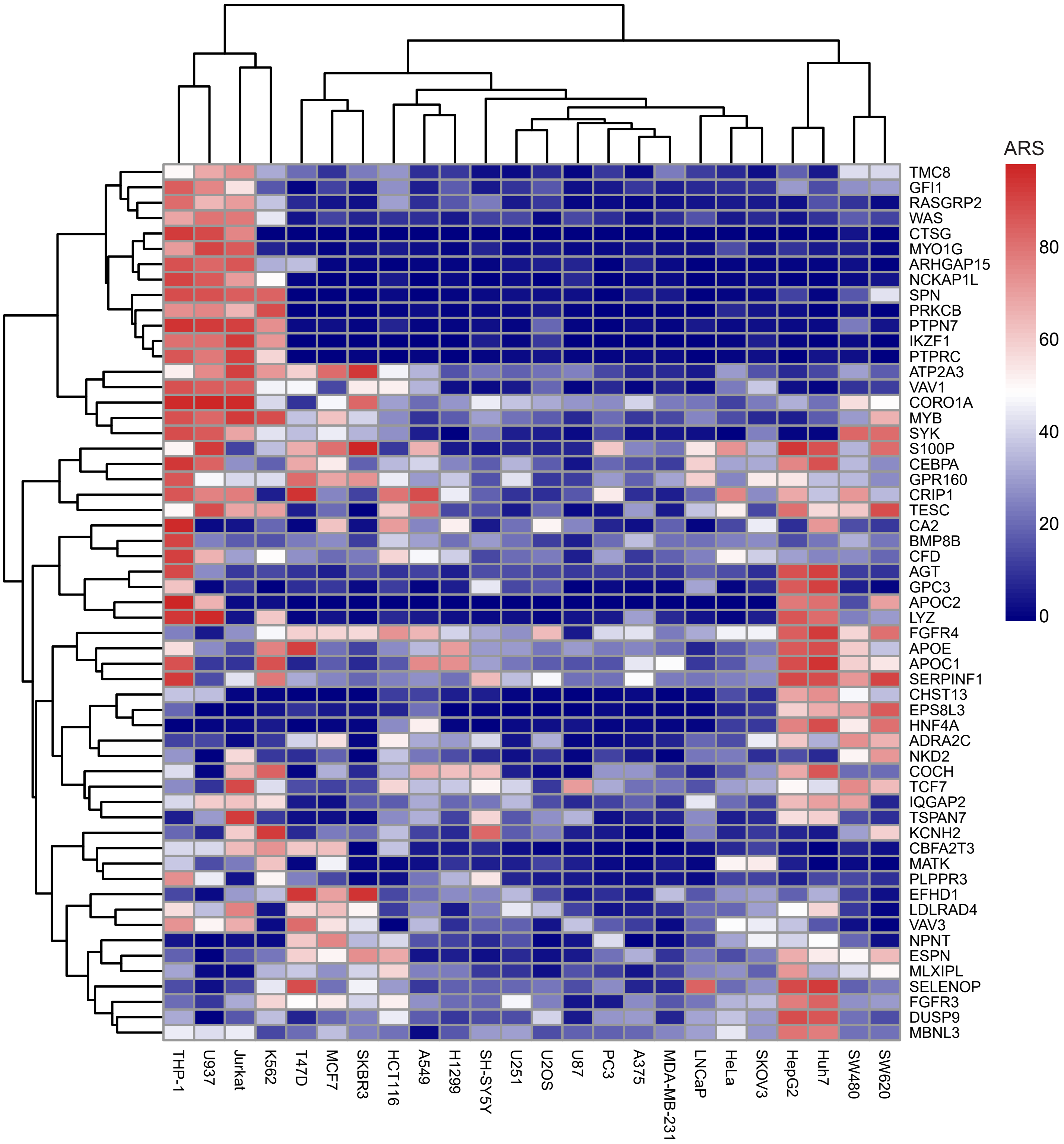

Figure S1. Average rank score (ARS) of 57 genes in cancer cell lines. The heatmap shows the ARS of the expression of these 57 genes in 24 cancer cell lines.

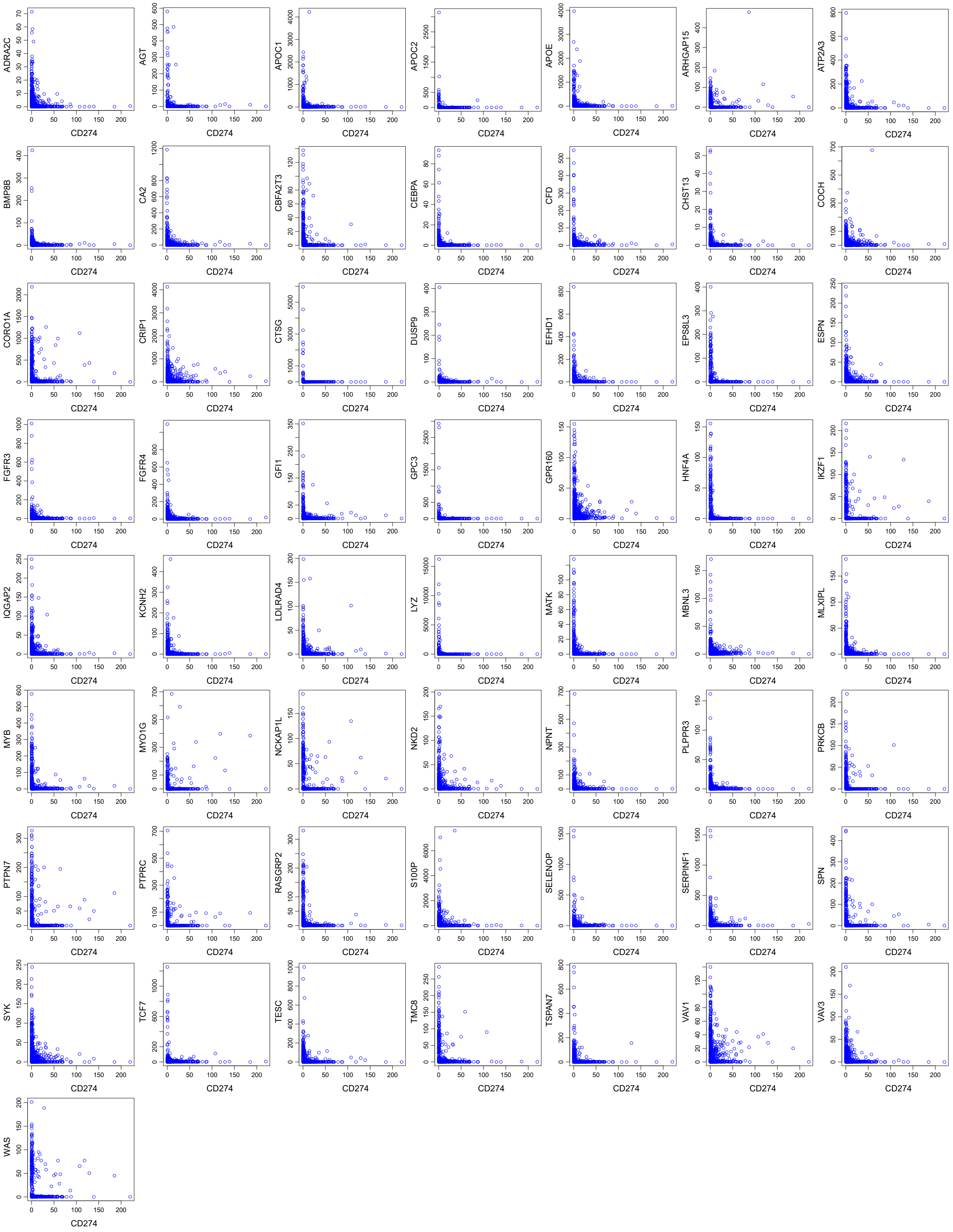

Figure S2. The mutual exclusion expression between genes and PD-L1 in CCLE samples. Scatter plots show the anti-correlated expression between 57 genes and PD-L1 expression, as derived from the CCLE database. The x-axis and y-axis represent the TPM values of CD274 and the 57 genes, respectively. Each point represents a cancer cell line.

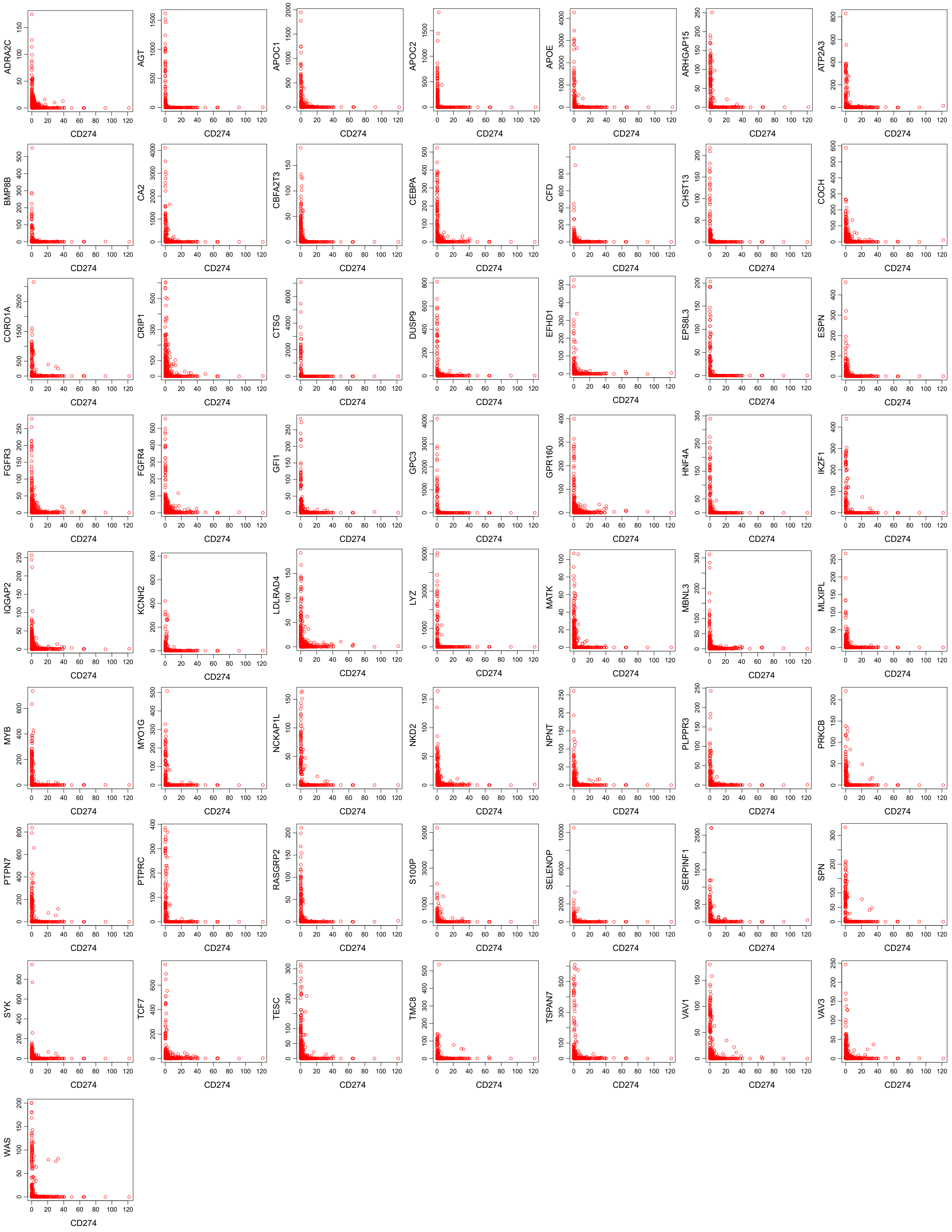

Figure S3. The mutual exclusion expression between genes and PD-L1 in RNA-Seq samples. Scatter plots show the anti-correlated expression between 57 genes and PD-L1 expression, as derived from present RNA-Seq dataset. The x-axis and y-axis represent the TPM values of CD274 and the 57 genes, respectively. Each point represents a sample.

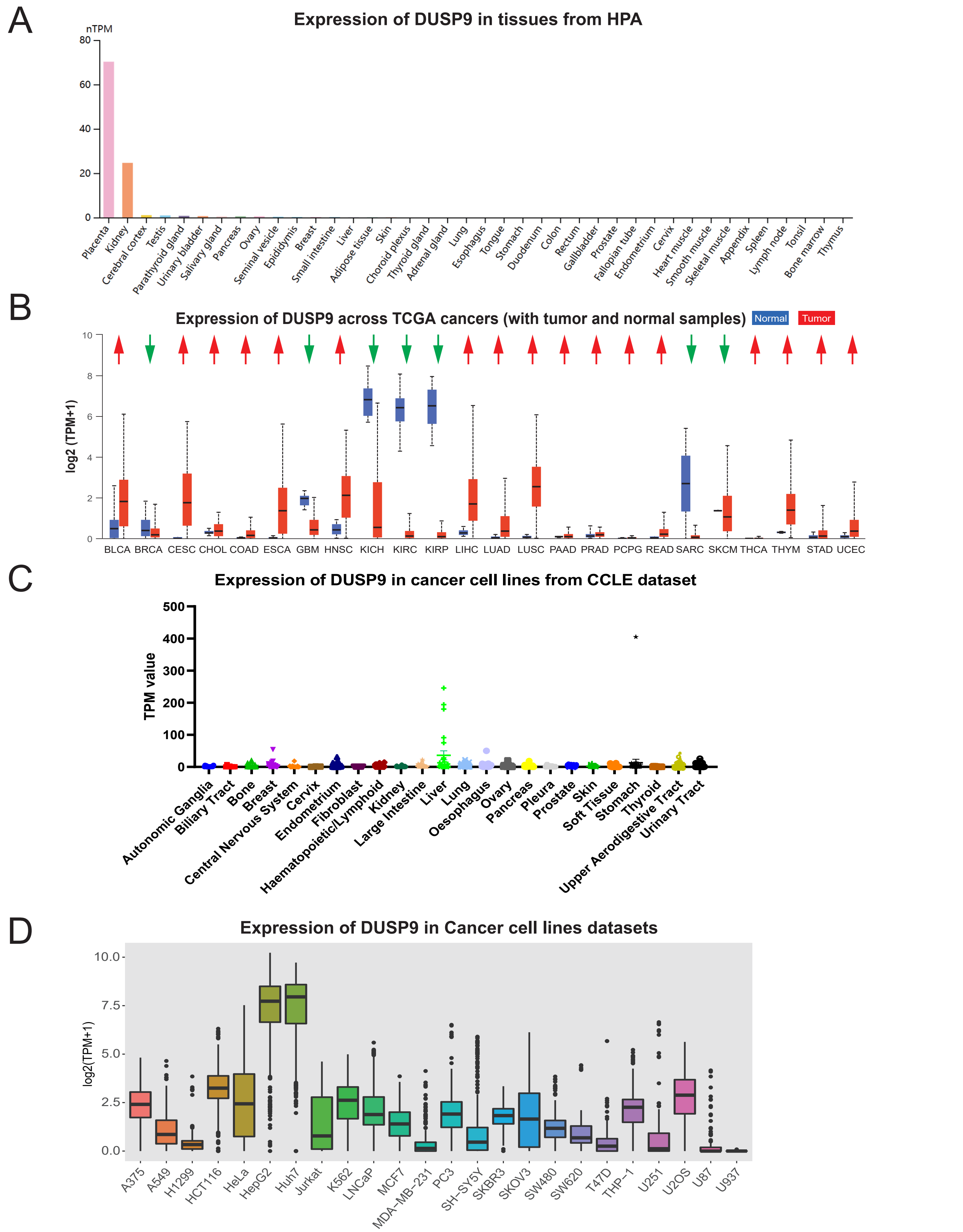

Figure S4. Expression of DUSP9 in tissues, tumors, and cancer cell lines. A) Expression levels of DUSP9 in tissues from the Human Protein Atlas (HPA). B) Expression levels of DUSP9 across TCGA cancers with tumor and normal samples retrieved from the University of Alabama at Birmingham Cancer (UALCAN) Data Analysis Portal (https://ualcan.path.uab.edu/).
C) Expression levels of DUSP9 in cancer cell lines from the CCLE dataset. Tumor cell lines are classified by tissue origin. Data are shown as mean ± SEM. D) Expression levels of DUSP9 in cancer cell lines from our cancer cell line datasets.

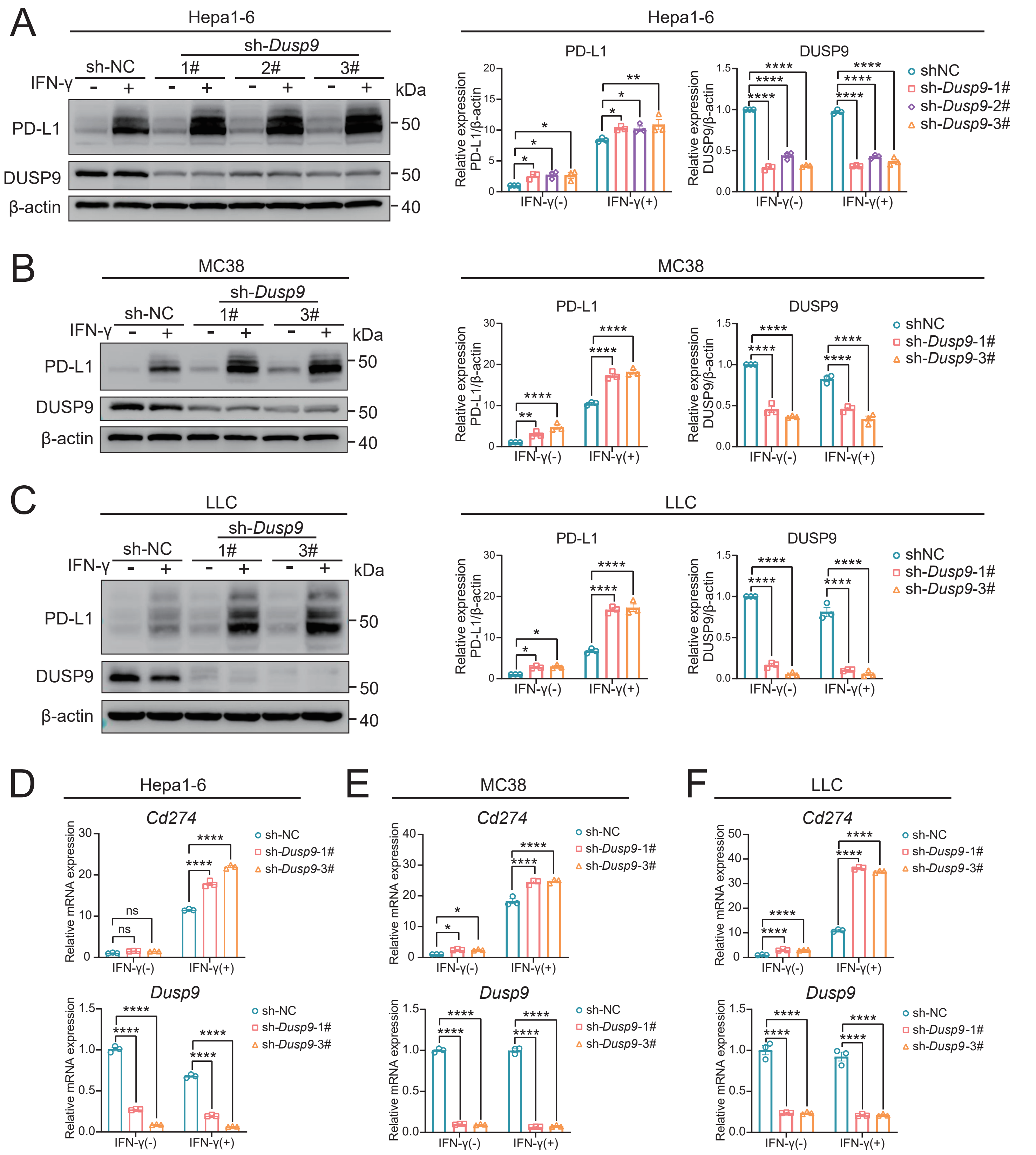

Figure S5. DUSP9 negatively regulates PD-L1 expression in mouse tumor cells. A-C) Western blot results show the expression of PD-L1 in DUSP9-KD Hepa1-6 cells (A), MC38 cells (B), and LLC cells (C). D-F) QPCR results show the mRNA expression of PD-L1 in DUSP9-KD Hepa1-6 cells (D), MC38 cells (E), and LLC cells (F). Data are shown as mean ± SEM; n = 3; *p* values were determined by two-way ANOVA with Dunnett's post-hoc test; ns, not significant; *, $p < 0.05$; **, $p < 0.01$; ****, $p < 0.0001$.

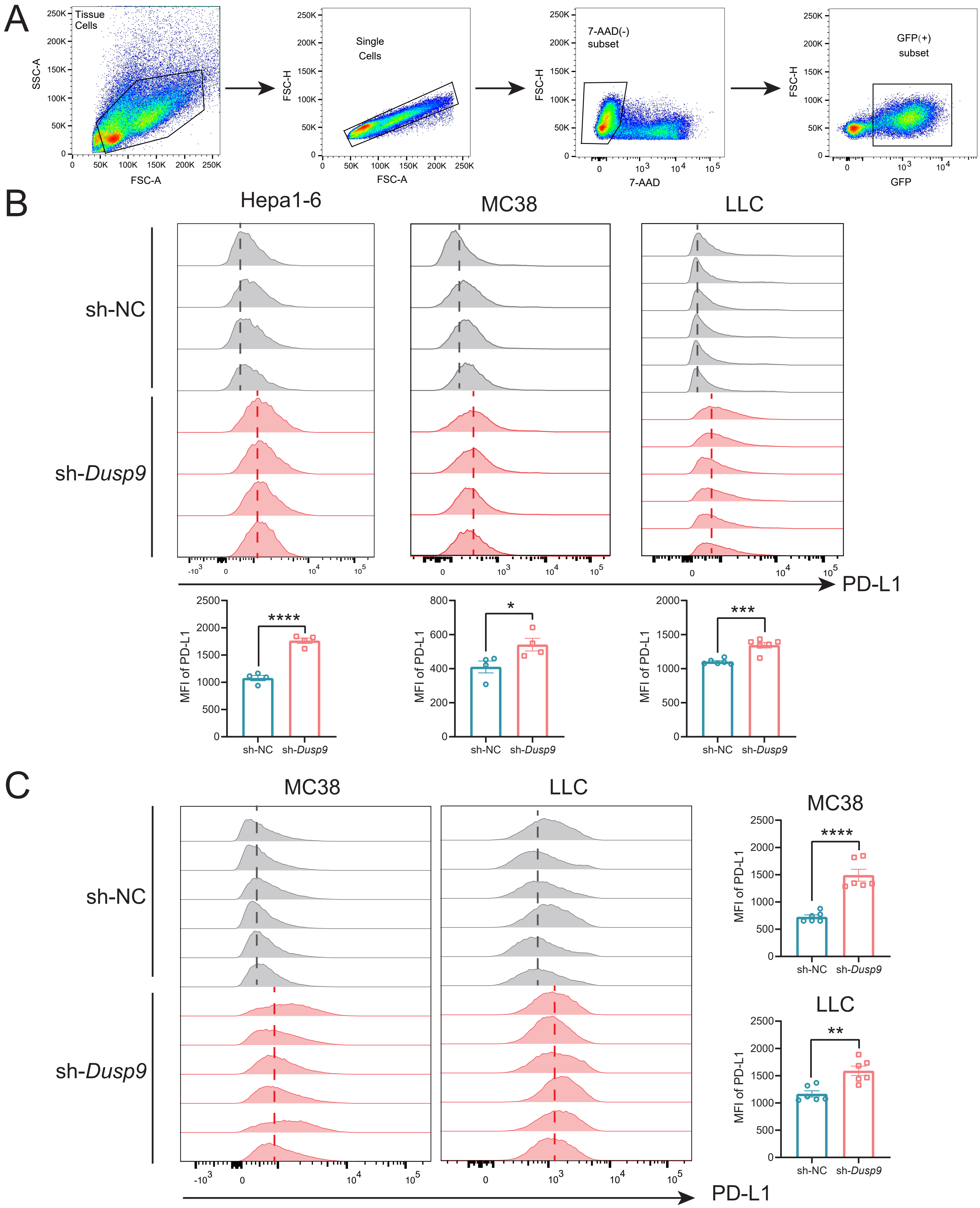

Figure S6. DUSP9 knockdown increases PD-L1 expression on the tumor cell surface in vivo. A) The gating strategy for the flow cytometry experiments. B) The results of the flow cytometry analysis of PD-L1 expression in tumor cells (GFP^+) derived from the DUSP9-KD Hepa1-6, MC38 and LLC tumor models in BALB/c nude mice. C) The results of the flow cytometry analysis of PD-L1 expression in tumor cells (GFP^+) derived from the DUSP9-KD MC38 and LLC tumor models in C57BL/6 mice. MFI, mean fluorescence intensity. Data are shown as mean ± SEM; n = 4 (Hepa1-6 and MC38 in BALB/c nude mice) or 6 (LLC in BALB/c nude mice, MC38 and LLC in C57BL/6 mice); *p* values were determined by Student's t-test; *, $p < 0.05$;**, $p < 0.01$; ***, $p < 0.001$; ****, $p < 0.0001$.

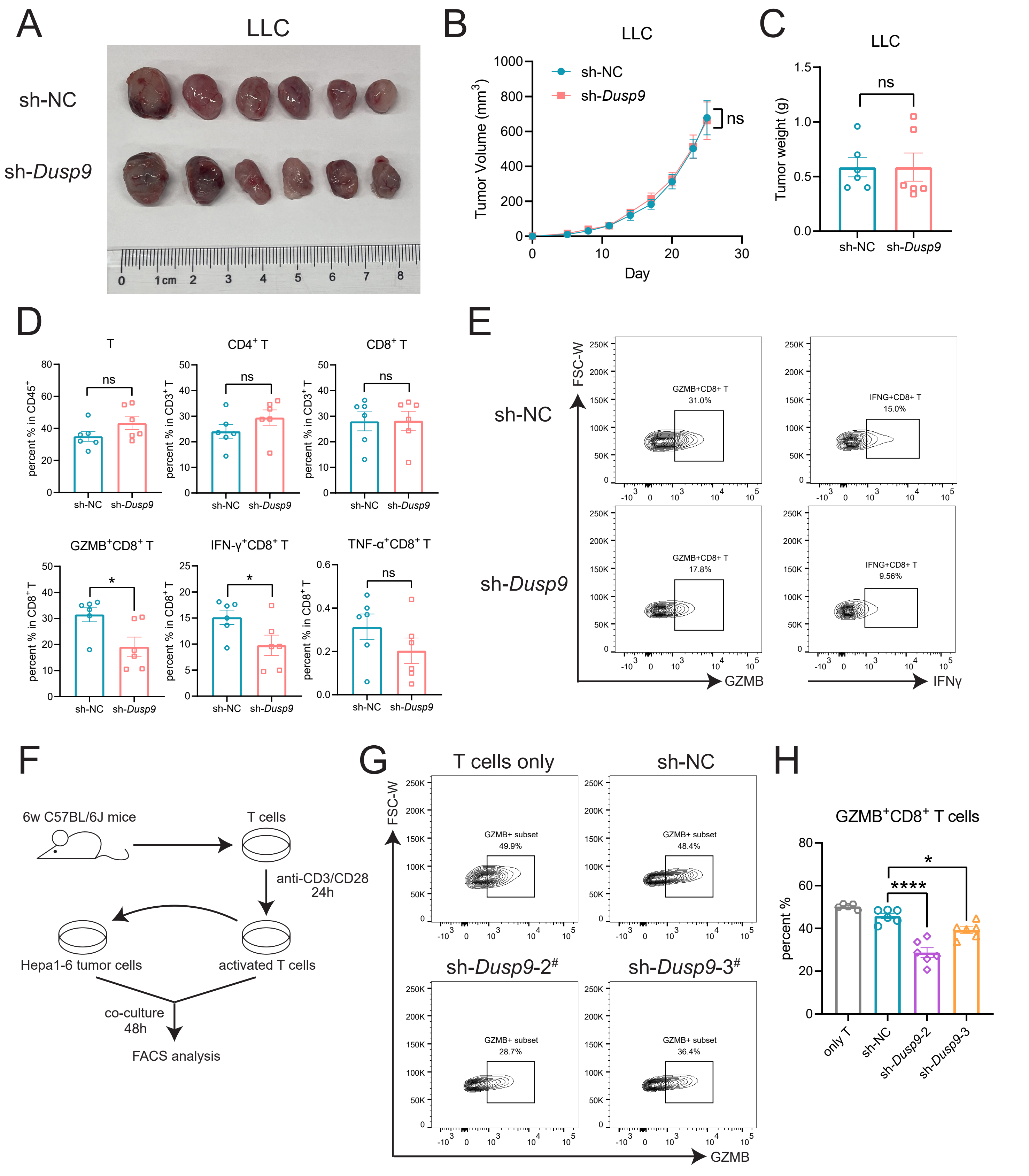

Figure S7. DUSP9 knockdown inhibits cytotoxic CD8$^+$ T cells. A-E) The syngenic tumor model of DUSP9-KD LLC cells in C57BL/6 mice includes the following: (A) tumor overview; (B) tumor growth; (C) tumor weight; (D) statistics on the percentage of tumor infiltrating T cell subsets; (E) GZMB$^+$CD8$^+$ T cell and TNF-α$^+$CD8$^+$ T cell subsets detected by flow cytometry. F-H) Co-culture of primary T cells and DUSP9-KD Hepa1-6 cells: (F) flowchart; (G) GZMB$^+$CD8$^+$ T cell subset detected by flow cytometry; (H) statistics on the percentage of the GZMB$^+$CD8$^+$ T cell subset. Data are shown as mean ± SEM; n = 6; *p* values were determined by Student's t-test (B-D) or one-way ANOVA with Dunnett's post-hoc test (H); ns, not significant; *, $p < 0.05$; ****, $p < 0.0001$.

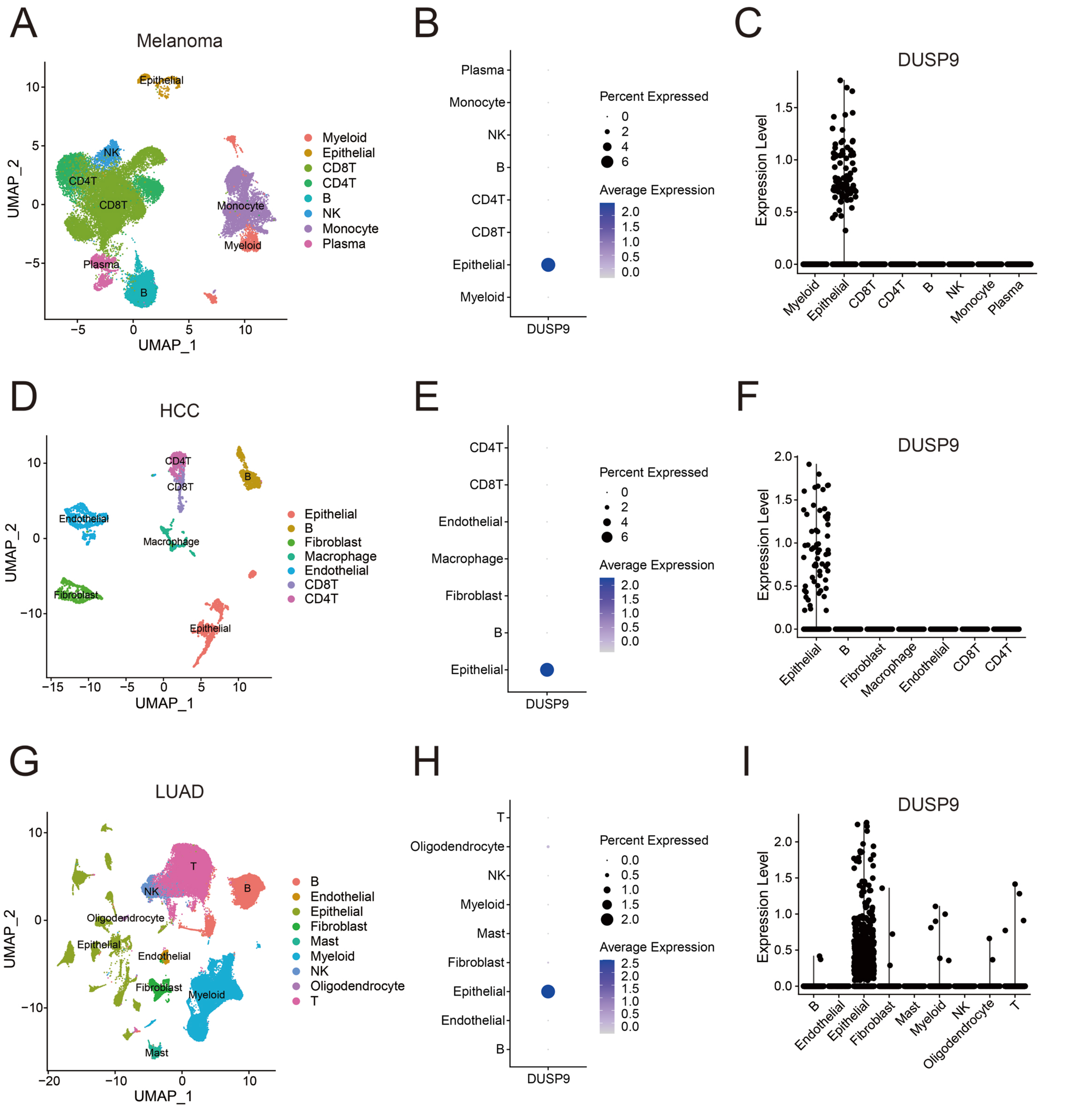

Figure S8. DUSP9 is mainly expressed in tumor cells based on single cell RNA-Seq data. A-C) Reanalysis of single cell RNA-Seq data from the GSE123139 dataset of human melanoma samples, including a Uniform Manifold Approximation and Projection (UMAP) reduction plot (A) for cell type annotation, a dot plot (B), and a scatter plot (C) for DUSP9 expression. D-F) Reanalysis of single cell RNA-Seq data from the GSE125449 dataset of human HCC samples, including a UMAP reduction plot (D) for cell type annotation, a dot plot (E) and a scatter plot (F) for DUSP9 expression. G-I) Reanalysis of single cell RNA-Seq data from the GSE131907 dataset of human lung adenocarcinoma (LUAD) samples, including a UMAP reduction plot (G) for cell type annotation, a dot plot (H) and a scatter plot (I) for DUSP9 expression.

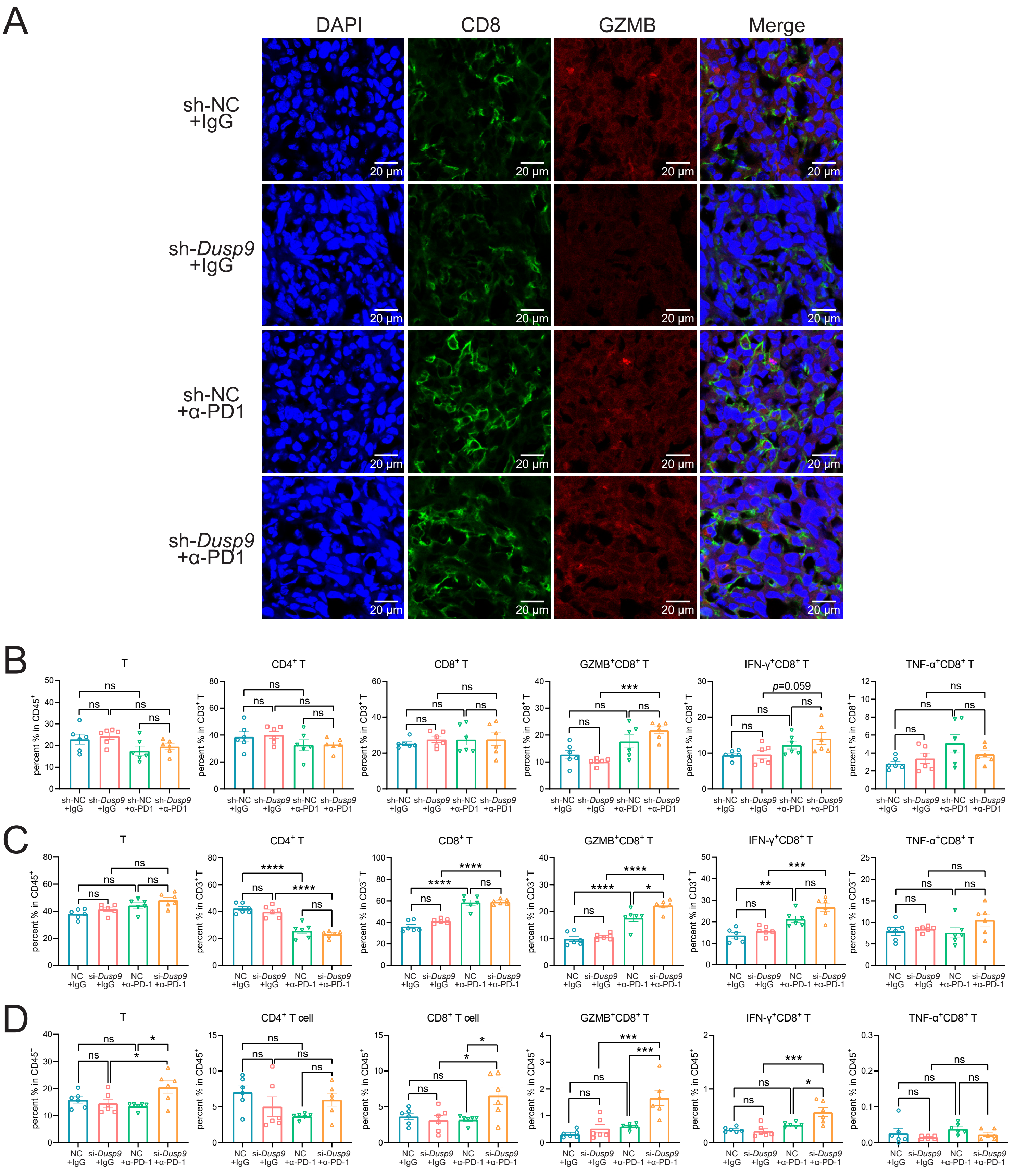

Figure S9. DUSP9 knockdown increased the infiltration of cytotoxic $CD8^+$ T cells following anti-PD-1 treatment. A) Immunofluorescence results show the expression of CD8 and GZMB in tumor tissues derived from C57BL/6 mice inoculated with DUSP9-KD MC38 cells and subsequently treated with anti-PD-1. B) Flow cytometry results show the proportion of T cells and T cell subgroups derived from C57BL/6 mice inoculated with DUSP9-KD LLC cells and subsequently treated with anti-PD-1. C-D) Flow cytometry results show the proportion of T cells and T cell subgroups derived from C57BL/6 mice inoculated with wild type MC38 cells (C) or LLC cells (D) and subsequently treated with siDusp9 and anti-PD-1. Data are shown as mean ± SEM; n = 6; p values were determined by one-way ANOVA with Sidak's post-hoc test; ns, not significant; *, $p < 0.05$; **, $p < 0.01$; ***, $p < 0.001$; ****, $p < 0.0001$.
