# Supplementary material for: Mutual Exclusion Analysis Shows that DUSP9 Negatively Regulates PD‐L1 Expression and Acts as a Target to Enhance Anti‐PD‐1 Efficacy
Source: Adv Sci (Weinh). 2025 Dec 17;13(12):e14347. doi: 10.1002/advs.202514347 (PMC12948242; doi:10.1002/advs.202514347)
Supplement: Supplementary file 4 — Supporting Information [file ADVS-13-e14347-s005.docx]

**Table S3. List of 600 RNA-Seq samples used for mutual exclusion analysis**

SRR10022355 SRR10029556 SRR10067385 SRR10081939 SRR10092105 SRR10098957 SRR10100509 SRR10102880 SRR10116230 SRR10116243 SRR10120327 SRR10139492 SRR10153183 SRR10153612 SRR10162417 SRR10168914 SRR10251244 SRR10259059 SRR10262680 SRR10262957 SRR10266097 SRR10291981 SRR10298073 SRR10319891 SRR10348341 SRR10368247 SRR10400227 SRR10402026 SRR10402228 SRR1042059 SRR10428385 SRR10434774 SRR10486789 SRR10497724 SRR10546865 SRR10546868 SRR1055402 SRR10559947 SRR10566929 SRR10587352 SRR10590689 SRR10603319 SRR10620039 SRR10620046 SRR10690979 SRR10721022 SRR10741163 SRR10755393 SRR10846495 SRR10850855 SRR10877209 SRR10897374 SRR10908767 SRR10969524 SRR10973967 SRR10990651 SRR11027012 SRR11027015 SRR11074202 SRR1107930 SRR11090002 SRR11124541 SRR11124570 SRR11124571 SRR11124603 SRR11124609 SRR11124625 SRR11182983 SRR11195269 SRR11213584 SRR11256498 SRR11281550 SRR11321432 SRR11321443 SRR11411722 SRR11412250 SRR11448363 SRR11452297 SRR11484250 SRR11496509 SRR11523083 SRR11538566 SRR11539212 SRR11539232 SRR11539441 SRR11579526 SRR11591057 SRR11591059 SRR11615323 SRR11615557 SRR11638703 SRR11647771 SRR11683658 SRR11683660 SRR11732461 SRR11772820 SRR11783870 SRR11788447 SRR11788477 SRR11788498 SRR11788559 SRR11790265 SRR1182617 SRR11830125 SRR11835777 SRR11841594 SRR11841598 SRR11856725 SRR11870125 SRR11870152 SRR11870155 SRR11870182 SRR11870193 SRR11870228 SRR11870235 SRR11870267 SRR11903031 SRR11920229 SRR11996357 SRR12010575 SRR12055591 SRR12082079 SRR12082091 SRR12082098 SRR12082143 SRR12090756 SRR12101916 SRR12147726 SRR12245098 SRR12248353 SRR12248358 SRR12248360 SRR12248361 SRR12248370 SRR12279057 SRR12279058 SRR12298104 SRR12298109 SRR12303191 SRR12316136 SRR12332564 SRR12332589 SRR12354633 SRR12391529 SRR12391535 SRR12412431 SRR12424175 SRR12450409 SRR12462460 SRR12462462 SRR1259319 SRR12616708 SRR12617208 SRR12637912 SRR12637921 SRR12637927 SRR12637968 SRR12637998 SRR12638006 SRR12638008 SRR12638029 SRR12638032 SRR12638047 SRR12638051 SRR12638057 SRR12638063 SRR12638070 SRR12739728 SRR12908230 SRR12979856 SRR1302911 SRR13036261 SRR13036591 SRR13150539 SRR13156939 SRR13263676 SRR13263722 SRR13263770 SRR13263807 SRR13267293 SRR13290039 SRR13290046 SRR13313970 SRR13321418 SRR13377364 SRR13393965 SRR13509051 SRR13600530 SRR13649912 SRR1374811 SRR13780407 SRR13927885 SRR13977853 SRR13977878 SRR14005509 SRR14094972 SRR14095565 SRR14218110 SRR14267821 SRR14346771 SRR14368749 SRR14436588 SRR14574921 SRR14664853 SRR14765834 SRR14917149 SRR15030357 SRR15183919 SRR1521490 SRR1521498 SRR1523611 SRR1523612 SRR1525259 SRR1554469 SRR15600644 SRR1640085 SRR1661554 SRR17167677 SRR17321370 SRR1767381 SRR1802128 SRR1910388 SRR1910389 SRR19142000 SRR1981231 SRR1981264 SRR1981289 SRR2001130 SRR2001147 SRR2016229 SRR2029840 SRR2044117 SRR2045037 SRR2049183 SRR2049190 SRR2056545 SRR2058411 SRR2060142 SRR2061442 SRR20761259 SRR20761260 SRR21091428 SRR2121674 SRR2135631 SRR21629265 SRR2184392 SRR21903226 SRR21922543 SRR2242942 SRR22577485 SRR23347363 SRR2473066 SRR2550622 SRR2569762 SRR2569861 SRR2569873 SRR2748142 SRR2846936 SRR2861526 SRR2924730 SRR2932382 SRR2936903 SRR3051733 SRR3070176 SRR3083565 SRR3096874 SRR3124502 SRR3130266 SRR3131354 SRR3240168 SRR3290965 SRR3337060 SRR3339206 SRR3340674 SRR3340706 SRR3350971 SRR3609582 SRR3624018 SRR3631716 SRR3643371 SRR3745018 SRR3764512 SRR3880269 SRR3932506 SRR3932637 SRR3933219 SRR3954208 SRR3987805 SRR3997492 SRR3997511 SRR4025933 SRR4044229 SRR4044235 SRR4191705 SRR4304719 SRR4304721 SRR4304732 SRR4340391 SRR4343895 SRR4343909 SRR4343932 SRR4343941 SRR4343947 SRR4343952 SRR4343987 SRR4344019 SRR4344021 SRR4344055 SRR4344076 SRR4344081 SRR4344091 SRR4348869 SRR4436506 SRR456526 SRR5006458 SRR5015886 SRR5027067 SRR5043784 SRR5052705 SRR5063982 SRR5125151 SRR5163966 SRR5168307 SRR5170595 SRR5170596 SRR5173295 SRR5183818 SRR5183832 SRR5188579 SRR5188583 SRR5223437 SRR5244287 SRR5268536 SRR5281779 SRR5296147 SRR5297169 SRR534301 SRR5345339 SRR5378434 SRR5416938 SRR5469251 SRR5469253 SRR5509285 SRR5574052 SRR5583295 SRR5583430 SRR5602683 SRR5659756 SRR5659782 SRR5659842 SRR5659845 SRR5659854 SRR5674284 SRR5674288 SRR5674302 SRR5735103 SRR5741948 SRR5749627 SRR5809425 SRR5809426 SRR5818003 SRR5821829 SRR5821830 SRR5839806 SRR5839971 SRR5859253 SRR5859260 SRR5877582 SRR5877628 SRR5877727 SRR5886649 SRR5886656 SRR5890855 SRR5928818 SRR5935013 SRR5935025 SRR5944079 SRR6024011 SRR6026765 SRR6031885 SRR6031886 SRR6039139 SRR6039147 SRR6048193 SRR6053473 SRR6061809 SRR6068900 SRR6078200 SRR6205342 SRR6216323 SRR6224350 SRR6286151 SRR6322524 SRR6337449 SRR6342244 SRR6342282 SRR6342287 SRR6342314 SRR6348980 SRR6348983 SRR6354159 SRR6380184 SRR6380186 SRR6440543 SRR6461133 SRR6501943 SRR653966 SRR6656708 SRR6661797 SRR6661799 SRR6666842 SRR6701596 SRR6746631 SRR6746635 SRR6796379 SRR6820621 SRR6822843 SRR6841403 SRR6841424 SRR6843216 SRR6847879 SRR6921693 SRR6921697 SRR6921713 SRR6945607 SRR6989630 SRR7003846 SRR7058055 SRR7071543 SRR7071548 SRR7089387 SRR7089422 SRR7120641 SRR7132483 SRR7132485 SRR7139930 SRR7142041 SRR7142049 SRR7146234 SRR7160980 SRR7203934 SRR7207298 SRR7240641 SRR7280707 SRR7280709 SRR7293198 SRR7343643 SRR7359678 SRR7359687 SRR7406437 SRR7413556 SRR7413557 SRR7440315 SRR7495295 SRR7499408 SRR7499415 SRR7499420 SRR7501009 SRR7521649 SRR7550532 SRR7586883 SRR7628252 SRR7647460 SRR7661337 SRR7687622 SRR7721738 SRR7798713 SRR7807583 SRR7807584 SRR7807585 SRR7865849 SRR7939366 SRR7939367 SRR7939375 SRR7939377 SRR7939693 SRR7947618 SRR8113018 SRR8133796 SRR8146613 SRR817000 SRR8187408 SRR8216503 SRR8242599 SRR8242611 SRR8274095 SRR8274237 SRR8274279 SRR8274294 SRR8274634 SRR8311492 SRR8330330 SRR8330331 SRR8330336 SRR8330338 SRR8375541 SRR8393123 SRR8423065 SRR8437844 SRR8444236 SRR8481943 SRR8481991 SRR8493677 SRR8554926 SRR8570628 SRR8570632 SRR8571092 SRR8574553 SRR8591254 SRR8592546 SRR8599089 SRR8599131 SRR8606153 SRR8634231 SRR8676841 SRR8676845 SRR8689203 SRR8695763 SRR8695773 SRR8695808 SRR8699607 SRR8699701 SRR8706189 SRR8706190 SRR8707446 SRR8707735 SRR8707740 SRR8743327 SRR8743332 SRR8743341 SRR8758369 SRR8769942 SRR8774304 SRR8848210 SRR8848224 SRR8858588 SRR8875232 SRR8875234 SRR8922884 SRR8980037 SRR8980050 SRR8985426 SRR8993405 SRR9005157 SRR9016986 SRR9016996 SRR9019154 SRR9029510 SRR9029988 SRR9042774 SRR9042779 SRR9042781 SRR9047216 SRR9082123 SRR9103047 SRR9108741 SRR9108755 SRR9110269 SRR9117121 SRR9180333 SRR9212556 SRR922112 SRR927169 SRR9310784 SRR9313013 SRR9604001 SRR9604051 SRR9604186 SRR9645575 SRR9672783 SRR9672794 SRR9697492 SRR9697503 SRR9698709 SRR9698761 SRR9698851 SRR9827847 SRR9829740 SRR9841045 SRR9861019 SRR9905403 SRR9905406 SRR9905425 SRR9905438 SRR9905520 SRR9905549 SRR9905552 SRR9905678 SRR9905795 SRR9905818 SRR9905832 SRR9905892 SRR9905896 SRR9905900 SRR9905905 SRR9906003 SRR9906020 SRR9906027 SRR9906106 SRR9922631
