# Supplementary material for: Mutual Exclusion Analysis Shows that DUSP9 Negatively Regulates PD‐L1 Expression and Acts as a Target to Enhance Anti‐PD‐1 Efficacy
Source: Adv Sci (Weinh). 2025 Dec 17;13(12):e14347. doi: 10.1002/advs.202514347 (PMC12948242; doi:10.1002/advs.202514347)
Supplement: Supplementary file 5 — Supporting Information [file ADVS-13-e14347-s003.docx]

**Table S4.** **The targeting sequences of short hairpin RNAs (shRNAs) and small interfering RNAs (siRNAs).**

| Name | Targeting gene symbol | Targeting sequence |
| --- | --- | --- |
| sh-*Dusp9*-1# | Mouse *Dusp9* | ccggCCTGTGCTTGAGCTCTGATTTCTC |
| sh-*Dusp9*-2# | Mouse *Dusp9* | ccggCTCACCTAACTTCAACTTTATCTC |
| sh-*Dusp9*-3# | Mouse *Dusp9* | ccggGCCCTTCTTGCTGTTTCATTTCTC |
| si-*CORO1A* | Human *CORO1A* | CCAUGACAGUGCCUCGAAATT |
| si-*FGFR4* | Human *FGFR4* | CUCGAAUAGGCACAGUUACTT |
| si-*KCNH2* | Human *KCNH2* | CCUUCGACCUGCUCAUCUUTT |
| si-*DUSP9*-1# | Human *DUSP9* | CGACUGCUCUGAUGCGGAATT |
| si-*DUSP9*-2# | Human *DUSP9* | CCAUUGAGUUCAUUGAUGATT |
| si-*STAT3* | Human *STAT3* | CACAAUCUACGAAGAAUCATT |
| si-*Dusp9* | Mouse *Dusp9* | GCAGAAGCUUCACCUCUCATT |
