# Supplementary material for: Mutual Exclusion Analysis Shows that DUSP9 Negatively Regulates PD‐L1 Expression and Acts as a Target to Enhance Anti‐PD‐1 Efficacy
Source: Adv Sci (Weinh). 2025 Dec 17;13(12):e14347. doi: 10.1002/advs.202514347 (PMC12948242; doi:10.1002/advs.202514347)
Supplement: Supplementary file 6 — Supporting Information [file ADVS-13-e14347-s004.docx]

**Table S5. The sequences of primers in real-time quantitative PCR (RT-qPCR).**

| Gene symbol | Forward primer sequence | Reverse primer sequence |
| --- | --- | --- |
| Human *CD274* | GGTGCCGACTACAAGCGAAT | AGCCCTCAGCCTGACATGTC |
| Human *CORO1A* | TGACACCAACATCGTCTACC | CTGGAACAGGTCCGACTTT |
| Human *DUSP9* | CAACCTCCCAAACTTCTTCG | ACAGTGACGGTGACAGAACG |
| Human *FGFR4* | TGCTATGCTAAACCTCCTGC | AACGCCATTTGCTCCTGT |
| Human *KCNH2* | CACCTAAGATAAAGGAGCGAA | TGTGAAGACAGCCGTGTAGA |
| Human *GAPDH* | GAAGGTGAAGGTCGGAGTC | GGAAGATGGTGATGGGATTT |
| Mouse *Cd274* | GCTCCAAAGGACTTGTACGTG | TGATCTGAAGGGCAGCATTTC |
| Mouse *Dusp9* | TGACCATTGGAGCCAGAAT | CATCGTTGAGTGAGAGGTGAA |
| Mouse *Actb* | GCAGCTCAGTAACAGTCCGC | AGTGTGACGTTGACATCCGT |
